# Interkingdom assemblages in human saliva display group-level surface mobility and disease-promoting emergent functions

**DOI:** 10.1073/pnas.2209699119

**Published:** 2022-10-03

**Authors:** Zhi Ren, Hannah Jeckel, Aurea Simon-Soro, Zhenting Xiang, Yuan Liu, Indira M. Cavalcanti, Jin Xiao, Nyi-Nyi Tin, Anderson Hara, Knut Drescher, Hyun Koo

**Affiliations:** ^a^Biofilm Research Laboratories, Department of Orthodontics, School of Dental Medicine, University of Pennsylvania, Philadelphia, PA 19104;; ^b^Division of Pediatric Dentistry, School of Dental Medicine, University of Pennsylvania, Philadelphia, PA 19104;; ^c^Division of Community Oral Health, School of Dental Medicine, University of Pennsylvania, Philadelphia, PA 19104;; ^d^Center for Innovation & Precision Dentistry, School of Dental Medicine and School of Engineering & Applied Sciences, University of Pennsylvania, Philadelphia, PA 19104;; ^e^Department of Physics, Philipps-Universität Marburg, 35032 Marburg, Germany;; ^f^Biozentrum, University of Basel, 4056 Basel, Switzerland;; ^g^Preventive and Restorative Sciences, School of Dental Medicine, University of Pennsylvania, Philadelphia, PA 19104;; ^h^Eastman Institute for Oral Health, University of Rochester Medical Center, Rochester, NY 14620;; ^i^Oral Health Research Institute, Department of Cariology, Operative Dentistry, and Dental Public Health, School of Dentistry, Indiana University, Indianapolis, IN 46202

**Keywords:** interkingdom interaction, microbial mobility, spatial structure, supraorganism, oral biofilm

## Abstract

Fungi and bacteria often engage in complex interactions, such as the formation of multicellular biofilms within the human body. Knowledge about how interkingdom biofilms initiate and coalesce into higher-level communities and which functions the different species carry out during biofilm formation remain limited. We found native-state assemblages of *Candida albicans* (fungi) and *Streptococcus mutans* (bacteria) with highly structured arrangement in saliva from diseased patients with childhood tooth decay. Further analyses revealed that bacterial clusters are attached within a network of fungal yeasts, hyphae, and exopolysaccharides, which bind to surfaces as a preassembled cell group. The interkingdom assemblages exhibit emergent functions, including enhanced surface colonization and growth rate, stronger tolerance to antimicrobials, and improved shear resistance, compared to either species alone. Notably, we discovered that the interkingdom assemblages display a unique form of migratory spatial mobility that enables fast spreading of biofilms across surfaces and causes enhanced, more extensive tooth decay. Using mutants, selective inactivation of species, and selective matrix removal, we demonstrate that the enhanced stress resistance and surface mobility arise from the exopolymeric matrix and require the presence of both species in the assemblage. The mobility is directed by fungal filamentation as hyphae extend and contact the surface, lifting the assemblage with a “forward-leaping motion.” Bacterial cell clusters can “hitchhike” on this mobile unit while continuously growing, to spread across the surface three-dimensionally and merge with other assemblages, promoting community expansion. Together, our results reveal an interkingdom assemblage in human saliva that behaves like a supraorganism, with disease-causing emergent functionalities that cannot be achieved without coassembly.

## Significance

Fungi and bacteria form multicellular biofilms causing many human infections. How such distinctive microbes act in concert spatiotemporally to coordinate disease-promoting functionality remains understudied. Using multiscale real-time microscopy and computational analysis, we investigate the dynamics of fungal and bacterial interactions in human saliva and their biofilm development on tooth surfaces. We discovered structured interkingdom assemblages displaying emergent functionalities to enhance collective surface colonization, survival, and growth. Further analyses revealed an unexpected group-level surface mobility with coordinated “leaping-like” and “walking-like” motions while continuously growing. These mobile groups of growing cells promote rapid spatial spreading of both species across surfaces, causing more extensive tooth decay. Our findings show multicellular interkingdom assemblages acting like supraorganisms with functionalities that cannot be achieved without coassembly.

The microbial life on Earth often resides on surfaces, where cells form multicellular structures known as biofilms (1). Extensive efforts have been devoted to understanding the biofilm formation process and the mechanisms underlying the biofilm lifestyle (1–3). While most studies have focused on bacteria, eukaryotic microbes also frequently form biofilms. Furthermore, previous studies have revealed that biofilms composed of bacteria and fungi are highly abundant in nature, establishing complex interkingdom interactions (4–7). Such bacterial–fungal biofilms can display enhanced virulence and survival, which is achieved through tight cell–cell cohesion, metabolite exchange, and extracellular polymeric matrices within established communities (4–6). How interkingdom biofilms initiate and develop on the surface, and which functions the different species carry out during this process, remains unclear.

In the human oral cavity, biofilms formed by bacteria and fungi have a major impact on health (7, 8). For example, patients affected by severe childhood caries (tooth decay), a widespread and costly infectious disease affecting toddlers worldwide (9), display high carriage of the bacterium *Streptococcus mutans* and the fungus *Candida albicans*, both in saliva and in biofilms formed on teeth (dental plaque) (10). Previous studies have shown that these distinct microbes form interkingdom biofilms with enhanced virulence under sugar-rich conditions (11, 12). However, interactions of these two species in saliva have not been characterized, and the extent to which the interactions between *S. mutans* and *C. albicans* influence the dynamics of biofilm formation and its functional properties is unknown.

In this study, we investigated the interactions between *S. mutans* and *C. albicans* during colonization and biofilm formation in human saliva, and made several unexpected discoveries with implications for disease. We observed that in saliva of toddlers affected by severe tooth decay, *S. mutans* and *C. albicans* formed highly structured interkingdom assemblages. Using real-time multiscale imaging and computational analysis, we studied the organization of such interkingdom assemblages and assessed their role during biofilm formation spatiotemporally. These experiments showed that bacterial clusters attached to yeast and hyphal complexes to form assemblages that display emergent properties, including enhanced surface colonization, a higher growth rate, and a stronger tolerance to shear stress and antimicrobials, which are not observed in either bacteria or fungi alone. Surprisingly, when individually tracked, these interkingdom assemblages display a unique mode of migratory group-level mobility, enabled by fungal filamentation across surfaces, which is used by the attached bacterial clusters for “hitchhiking.” Through this mobility, the interkingdom assemblages rapidly proliferate across the surface and expand three-dimensionally, leading to biofilm superstructures and extensive enamel decay on ex vivo tooth surfaces that cannot be achieved by each species alone. Hence, our data reveal an interkingdom assemblage found in human saliva that efficiently colonizes, displays emergent properties, and enhances surface spreading through a group-level mobility mechanism that propels clusters of otherwise nonmotile bacteria and fungi across the surface, to ultimately promote community spatial expansion and disease-causing activity.

## Results

### Interkingdom Microbial Assemblages Occur in Human Saliva from Childhood Caries Patients.

**Fig. 1.**
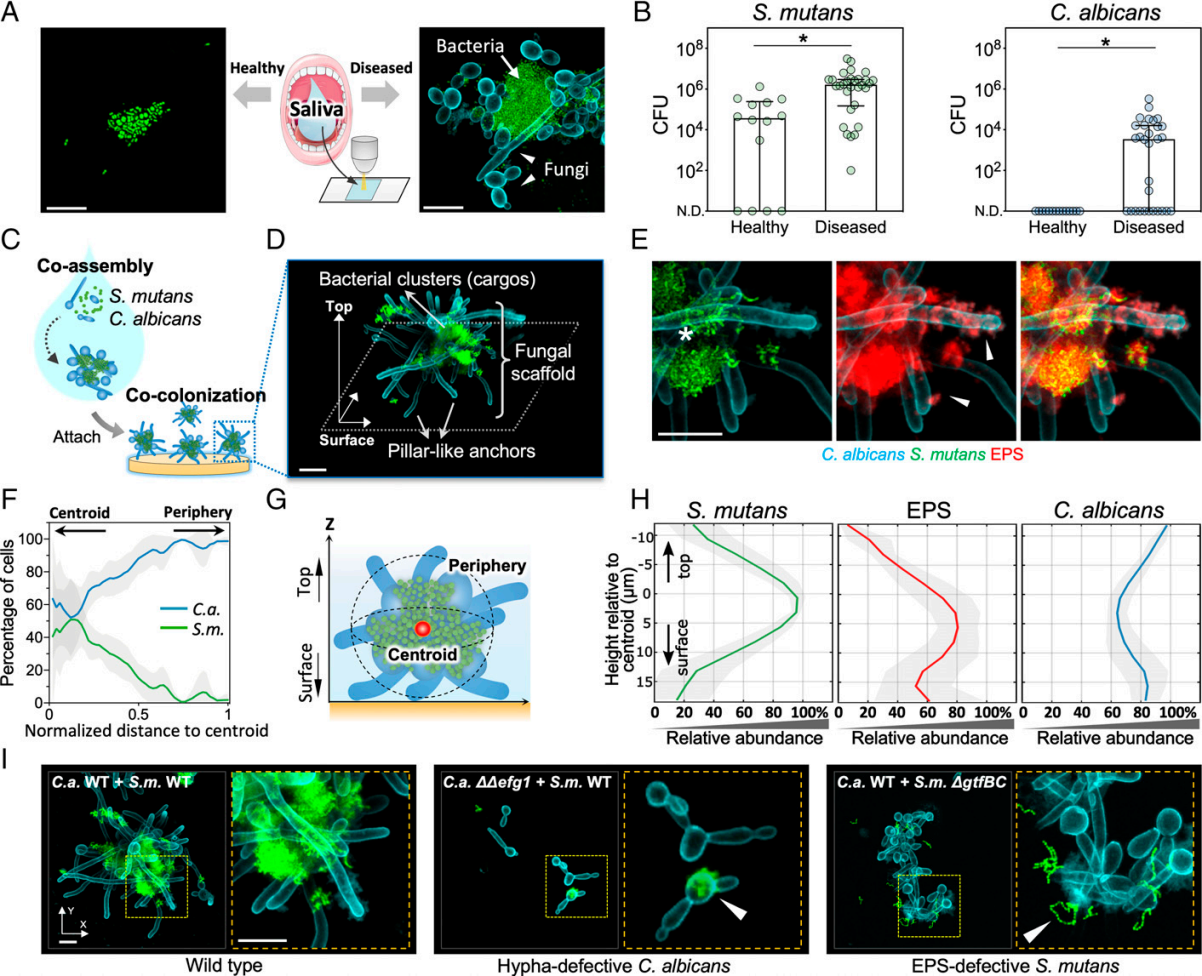
Interkingdom microbial assemblages in saliva attach to surfaces as structured cell groups. (*A*) Using fluorescent staining and confocal microscopy, native fungal–bacterial assemblages are found in saliva of patients with early childhood caries, but not in healthy individuals. (*B*) *S. mutans* and *C. albicans* were found in high levels in the diseased plaque. Data are presented as median with interquartile range. *P < 0.05 by Mann–Whitney *U* test. (*C*) Interkingdom assemblages and surface colonization is recapitulated using an in vitro model based on human saliva and hydroxyapatite surfaces, to mimic the tooth enamel. (*D*) Spatially structured *S. mutans* and *C. albicans* assemblage on the tooth-mimetic surface. (*E*) Surface-colonized assemblage using different fluorescent markers, as indicated underneath the images. EPS: extracellular α-glucan matrix produced by *S. mutans*. Asterisk: inner core harboring a mix of *C. albicans* and *S. mutans*. Arrowheads: peripheral areas containing mostly *Candida* hypha covered with bacterial-derived EPS. (*F*) Spatial distribution of *S. mutans* and *C. albicans* within the assemblage. Lines correspond to mean and shaded region to SD of *n* = 4 independent replicates. (*G*) Schematic diagram describing the spatial arrangement of the two species inside the assemblage, based on the computational image analysis results from *F* and *H*. (*H*) Spatial organization of the fungal and bacterial species relative to the surface. Lines correspond to mean and shaded area to SD of *n* = 4 independent replicates. (*I*) Confocal images of initial surface colonizers, for WT–WT assemblages and different WT–mutant assemblages (using *C. albicans ΔΔefg1* or *S. mutans ΔgtfBC* mutants). (Scale bars, 10 μm.)

We collected saliva from healthy (caries-free) children and children with severe childhood caries and analyzed the intact, naturally present microbial content by fluorescence in situ hybridization (FISH) and super resolution confocal imaging (Fig. 1*A*). We found that saliva from diseased patients was enriched with assemblages of fungal and bacterial cells. In these assemblages, bacterial clusters were physically associated with fungal cells (yeasts and hyphae/pseudohyphae) forming a multicellular structure (Fig. 1 *A*, *Right*). In contrast, saliva from healthy (caries-free) children contained mostly single-cell bacteria or bacterial aggregates (Fig. 1 *A*, *Left*). To determine the species composition of the assemblage, we performed FISH imaging using species-specific probes, noting that early childhood caries patients typically harbor high levels of *C. albicans* and *S. mutans* in saliva (9, 10). We found that interkingdom assemblages in saliva from early childhood caries patients were comprised primarily of *C. albicans* and *S. mutans* (*SI Appendix*, Fig. S1). We also detected α-glucans, an extracellular polysaccharide (EPS) associated with tooth-decay (13). α-Glucans are produced predominantly by *S. mutans*-derived exoenzymes termed glucosyltransferases, or Gtfs (14). We observed α-glucans on the bacterial and fungal cell surfaces within the interkingdom assemblage in saliva from the diseased patients (*SI Appendix*, Fig. S2*A*). We assessed Gtf activity using radiolabeling and scintillation counting and found higher Gtf activity levels in the saliva from diseased children (vs. healthy children) (*SI Appendix*, Fig. S2*B*). In the diseased plaque biofilm, both *C. albicans* and *S. mutans* were found in high levels (Fig. 1*B*), but significantly less in healthy samples, suggesting a dynamic interaction of *S. mutans* and *C. albicans* as they transition from the fluid phase to an apatitic surface.

These results from patient samples show that interkingdom assemblages in saliva constitute a complex biostructure of bacteria, fungi, and EPS α-glucans, which cooccurs with early childhood caries. The influence of these interkingdom assemblages on tooth surface colonization and biofilm formation, and the properties of these assemblages, are investigated in this study, as described below.

### Experimental Model for Interkingdom Assemblage and Surface Colonization in Saliva.

To investigate the assemblages of *S. mutans* and *C. albicans* in more detail, we sought to recreate such interkingdom assemblages in the laboratory. We therefore developed an experimental model using *C. albicans* and *S. mutans* incubated in human saliva at 37 °C, and hydroxyapatite as a tooth-mimetic surface (Fig. 1*C*). In this model system, we found that planktonic *C. albicans* and *S. mutans* can coassemble to form assemblages with similar structural features to those naturally present in the patients’ saliva, which are characterized by yeast and hyphal forms intertwined with *S. mutans* clusters and EPS α-glucans (*SI Appendix*, Fig. S3). We then assessed whether these bacterial-fungal assemblages can bind to saliva-coated hydroxyapatite (sHA) surfaces. We found that these assemblages, formed in the saliva prior to surface contact, attached to the sHA surface as a cell group (*SI Appendix*, Fig. S4*A*). These findings were further corroborated by culturing the viable cells recovered from sHA surfaces, which revealed higher counts of *S. mutans* and *C. albicans* when they were incubated together in saliva (vs. each alone) (*SI Appendix*, Fig. S4*B*), indicating that coassembly may benefit both species for enhanced surface binding. Three-dimensional (3D) reconstruction of the confocal fluorescence images showed a network of *C. albicans* yeast cells and hyphae harboring *S. mutans* within the assemblage structure, which attaches to the sHA surface as a group (Fig. 1*D*; individual fluorescence channels shown in *SI Appendix*, Fig. S5*A*). The 3D images showed fungal hyphae located at the periphery adhering to the surface in a pillar-like arrangement, whereas most of the bacterial cells were clustered and attached onto the fungal surface like “cargo” (*SI Appendix*, Fig. S5).

Next, we employed computational image analysis (15) to investigate the composition and the spatial structuring of the microbial and EPS components within the interkingdom assemblages. We found that the core of the assemblage, in the vicinity of the center-of-mass (referred to as “centroid”), harbored a mix of *C. albicans* and *S. mutans*, whereas the periphery of the assemblage was predominantly comprised of *C. albicans* hyphae (close-up shown in Fig. 1 *E*, *Left*; quantification in Fig. 1*F*; schematic diagram in Fig. 1*G*). A schematic diagram depicting the location of the centroid and periphery of the assemblage as well as the surface is shown in Fig. 1*G*. Notably, fungal cells localized across the entire structure including the surface-contacting areas (Fig. 1 *H*, *Right*). In contrast, bacteria localized mostly around the inner core, close to the centroid, which is significantly above the surface (Fig. 1 *H*, *Left*). These results for the bacterial and fungal organization in the interkingdom assemblages are summarized in Fig. 1*G*. Matching the location of the bacterial clusters, EPS α-glucans were detected both in the core of the assemblage and also at peripheral locations across the hyphae surface (Fig. 1 *E*, *Center* and *Right*, and Fig. 1 *H*, *Center*). This is consistent with previous reports indicating that Gtf exoenzymes (which produce α-glucans) can bind to both bacterial and fungal cell membranes to mediate bacterial clustering and in situ glucans synthesis on the *C. albicans* surface (16).

Given the spatial location of hyphal cells and EPS, we hypothesized that fungal hyphal formation, adhesins, and streptococcal Gtfs (12, 17) are key factors for the interkingdom assemblage and colonization. To test this hypothesis, we initially used *C. albicans* mutants in which core transcriptional regulators associated with hyphal formation were deleted (18), to determine their impact on the multicellular structure (*SI Appendix*, Fig. S6). Among *Candida* mutants, we found that the *efg1* knockout strain, a master regulator of *C. albicans* hyphal formation, was most disruptive to assemblage formation, and resulted in only a sparse colonization of mostly single cells of bacteria and fungi on the sHA surface (vs*. C. albicans* WT strain) (Fig. 1*I* and *SI Appendix*, Fig. S6*A*). We next analyzed *C. albicans* Efg1-regulated adhesins expressed on the hyphal cell wall using homozygous knockout strains, including *ΔΔals1/ΔΔals3*, *ΔΔhwp2*, *ΔΔhyr1*, and *ΔΔeap1* (*SI Appendix*, Fig. S6*A*). Since Als1 and Als3 have a substantial overlap in functions (19), we used a double mutant with a disruption of both genes. Similar to the *efg1* knockout, the *ΔΔals1/ΔΔals3* deletion caused a severe reduction of coassembly, whereas *ΔΔhwp2*, *ΔΔhyr1*, and *ΔΔeap1* did not show a significant impact (*SI Appendix*, Fig. S6*A*). Furthermore, *ΔΔals1/ΔΔals3* also led to a significant disruption of surface colonization, resulting in few cells adhered on the surface (*SI Appendix*, Fig. S6*A*). Conversely, we investigated whether the Gtf-derived EPS contributes to this process. Using a *S. mutans* double-knockout of *gtfB* and *gtfC*, we found that interkingdom assembly was also disrupted harboring mostly single-chain bacterial cells (Fig. 1*I* and *SI Appendix*, Fig. S6*B*). This finding was further confirmed by adding glucanohydrolases (20) to the saliva exogenously, which specifically break down the α-glucans produced by *S. mutans* GtfB and GtfC (*SI Appendix*, Fig. S6*B*). Moreover, in the absence of sucrose (the substrate for EPS α-glucans synthesis by Gtf enzymes), the ability of *C. albicans* and *S. mutans* to colonize as structured interkingdom assemblages was impaired (*SI Appendix*, Fig. S7). These results indicate that Efg1-regulated *Candida* hyphal formation, hypha-specific Als adhesins, and streptococcal Gtf-derived α-glucans play important roles for the formation of interkingdom assemblages and for the surface colonization by these multicellular biostructures.

Altogether, the experiments in our saliva-based biofilm model showed that *C. albicans* and *S. mutans* can coassemble in saliva into a structured interkingdom assemblage, which enables enhanced colonization of both species on tooth-mimetic surfaces. Analysis of the spatial organization of the interkingdom assemblages revealed three features: 1) *C. albicans* hyphae are located at the periphery and hyphal surface contacts may serve as anchors; 2) Surface colonization of *S. mutans* clusters is promoted by attachment to the fungal network, which carries the bacterial clusters like cargo; and 3) hyphae formation and the presence of EPS α-glucans are both critical for the assemblage formation and surface colonization.

### Interkingdom Assemblages Display Enhanced Mechanical Resistance and Antimicrobial Tolerance.

The coassembled fungi and bacteria can colonize the surface as a highly structured group, which may confer additional advantages under various challenges. We examined whether these interkingdom assemblages withstand mechanical shear stress generated by fluid flow, and their susceptibility to antimicrobial treatment.

**Fig. 2.**
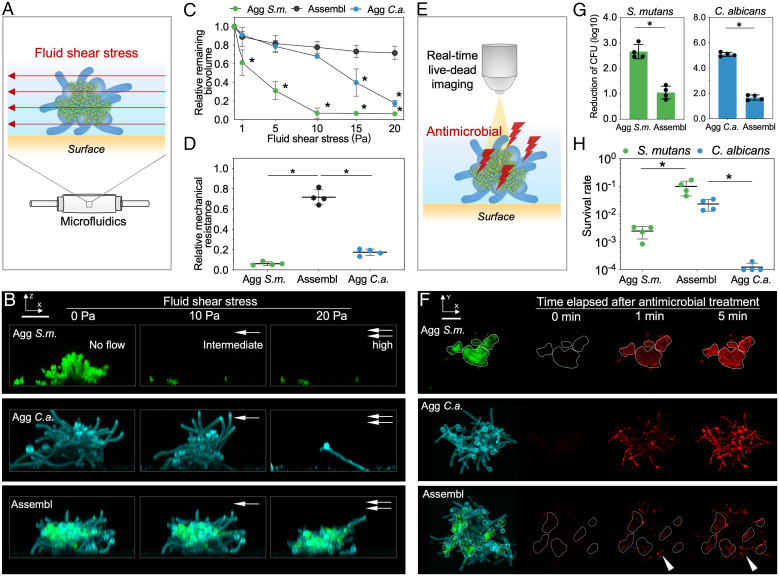
Enhanced mechanical resistance and antimicrobial tolerance of surface-attached interkingdom assemblage. (*A*) Schematic diagram of the microfluidic device used to test the mechanical resistance of surface-attached assemblages. (*B*) Time-lapse confocal imaging of the cellular response to the surface detachment force. *Left* image (0 Pa) illustrates surface-attached biostructures prior to shear stress exposure. *Center* and *Right* images show the same biostructures after being exposed to intermediate (10 Pa) or high shear stress (20 Pa). Green, *S. mutans*; cyan, *C. albicans*. (Scale bar, 20 μm.) (*C*) Relative remaining biovolume of the surface-attached biostructures after applying different shear stress. For interkingdom assemblages, the total biovolume of *S. mutans* and *C. albicans* was calculated. (*D*) Relative mechanical resistance of surface-attached assemblages after exposed to high shear stress (20 Pa), which is defined as the ratio of remaining biovolume on the surface to the original biovolume. (*E*) Schematic diagram of the experimental setup for the real-time antimicrobial killing assay for surface-attached assemblages, using 100 µg/mL chlorhexidine. (*F*) Time-lapse confocal image of antimicrobial killing. Dead cells (bacteria and fungi) are visualized using Toto-3 iodide (in red). *Left* image illustrates biostructures prior to antimicrobial exposure. Green, *S. mutans*; cyan, *C. albicans*. Images on the *Right* show the real-time killing profile (red channel only) within the same surface-attached biostructure. White solid lines indicate the bacterial clusters within aggregated *S. mutans* or interkingdom assemblage. Green, *S. mutans*; cyan, *C. albicans;* red, dead cells (*S. mutans* and *C. albicans*). (Scale bar, 20 μm.) (*G*) Reduction of S. *mutans* and *C. albicans* CFU in different biostructures after 5-min antimicrobial treatment. (*H*) Survival rate for each species in the biostructures after 5-min treatment. Abbreviation: Agg *C.a.*, aggregated *C. albicans*; Agg *S.m.*, aggregated *S. mutans*; Assembl, interkingdom assemblages. *P < 0.05 by one-way analysis of variance with Dunnett's multiple-comparison test (*C* and *D*) or by Student’s *t* test (*G* and *H*).

After attachment of the interkingdom assemblages to sHA surfaces, we applied increasing shear stress ranging from 1 to 20 Pa and assessed the detachment of the assemblages in real-time using confocal live-cell imaging (Fig. 2*A*). We found that surface-colonized *S. mutans* or *C. albicans* alone were dissembled and readily detached from the sHA surface under increased fluid shear stress. In contrast, the interkingdom assemblage remained attached to the surface, maintaining its structural stability even under high shear stress (Fig. 2*B*). Quantitative analysis revealed distinctive detachment patterns (Fig. 2*C*). Most of the aggregated *S. mutans* cells detached from the sHA surface at low to intermediate shear stress (>90% removal at 10 Pa). Notably, aggregated *C. albicans* could withstand intermediate shear stress levels (<30% removal at 10 Pa), but were removed when shear stress continued to increase (>80% removal at 20 Pa). In contrast, most of the assemblages remained on the surface under even the highest shear stresses (<30% removal at 20 Pa), displaying several-fold higher resistance to physical clearance compared to either species alone (Fig. 2*D*). These data show that the formation of an interkingdom assemblage provides enhanced mechanical stability to both species.

Next, we assessed the susceptibility of the interkingdom assemblages to antimicrobials. By analyzing spatiotemporal killing dynamics following exposure to chlorhexidine (Fig. 2*E*), a commonly used antimicrobial agent in mouthwash, we detected killing of bacterial or fungal cells alone within 1 min (Fig. 2*F*). This finding shows that cells located within the monospecies aggregates of either species were rapidly killed by the antimicrobial agent. In contrast, the interkingdom assemblages were remarkably tolerant to chlorhexidine, and only a small portion of cells, mostly located in the outer layers of the assemblage (white arrowhead in Fig. 2*F*), were killed even after 5 min. Furthermore, viable cell counting confirmed that both species in the assemblage displayed a higher antimicrobial tolerance (Fig. 2*G*), resulting in higher survival rate (Fig. 2*H*).

Given the availability of narrow-spectrum antifungals, we also tested whether *C. albicans* residing in the assemblage displayed enhanced resistance against nystatin, a fungicide that has no antibacterial effect on *S. mutans* (21). We found a similar killing pattern whereby nystatin was unable to effectively kill the fungal cell within the assemblage (*SI Appendix*, Fig. S8).

Since the cocolonized bacterial and fungal cells are enmeshed by EPS α-glucans (Fig. 1*E*), we assessed whether α-glucan degradation using exogenously supplied glucanohydrolases (dextranase and mutanase) could impact both the mechanical and antimicrobial resistance. For these assays, we used a localized, brief pretreatment method that degrades the α-glucans without biocidal activity while preserving the overall 3D structure of the interkingdom assemblage (20). The data show that the surface-attached biostructure was readily removed by increased fluid shear after pretreatment with glucanohydrolase (>90% removal at 20 Pa) (*SI Appendix*, Fig. S9), suggesting that EPS degradation weakened its attachment strength. We then examined whether EPS-degradation could affect the antimicrobial tolerance against both chlorhexidine (*SI Appendix*, Fig. S10) and nystatin (*SI Appendix*, Fig. S8). We found that the microbes were more effectively and homogeneously killed across the entire interkingdom assemblage after pretreatment with glucanohydrolase, indicating a protective role provided by the locally produced α-glucans within the interkingdom assemblage.

We also assessed whether the emergent properties are species-specific. We used *Streptococcus gordonii*, an early-colonizer oral commensal species (13), to form interkingdom assemblages with *C. albicans*. We found that the surface-attached *C. albicans*–*S. gordonii* assemblages were readily removed by increased fluid shear (>85% removal at 10 Pa) (*SI Appendix*, Fig. S9), suggesting much weaker attachment strength vs. the *C. albicans–S. mutans* assemblage. We also observed that *C. albicans* and *S. gordonii* within the assemblage were rapidly killed by chlorhexidine contrasting with the enhanced antimicrobial tolerance of *C. albicans*–*S. mutans* assemblage (*SI Appendix*, Fig. S10). The data suggest bacterial species-specificity associated with emergent properties displayed by the interkingdom assemblage.

Taking these data together, we find that by cocolonizing as an interkingdom assemblage, *C. albicans* and *S. mutans* display enhanced tolerance against mechanical clearance and antimicrobial compounds, which promote the persistence and survival of these biostructures that are acting as initial colonizers of surfaces. Importantly, these benefits were not observed in monospecies aggregates, indicating a highly interdependent partnership between bacterial and fungal cells.

### Surface-Bound Interkingdom Assemblages Grow Faster and Initiate Biofilms.

**Fig. 3.**
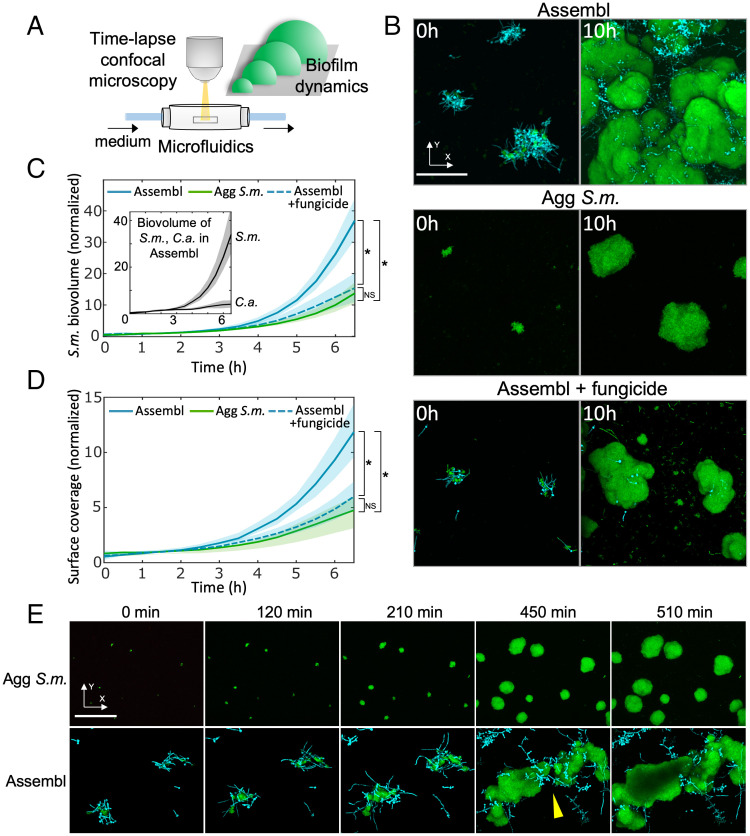
Biofilm growth dynamics of interkingdom assemblages on tooth-mimetic surface. (*A*) Schematic diagram of the flow-cell microfluidic culture system coupled with time-lapse confocal microscopy for visualizing biofilm growth. (*B*) Confocal images of the initial colonizers on the surface at 0 h, and the biofilm structure after 10 h, for interkingdom assemblage (Assembl), for aggregated *S. mutans* (Agg *S.m.*), and for fungicide-treated assemblage (250 µg/mL nystatin for 30 min). Green, *S. mutans*; cyan, *C. albicans*. (Scale bar, 100 μm.) (*C*) Time-resolved biofilm biovolume of *S. mutans* during the biofilm development. (*Inset*) Time-resolved biovolume of each *S. mutans* and *C. albicans* within the assemblage. Lines correspond to mean, shaded region to SD of *n* = 4 independent replicates. (*D*) Quantification of the dynamics of biofilm surface spreading. Lines correspond to mean, shaded region to SD of *n* = 4 independent replicates. *P < 0.05 by one-way analysis of variance with Tukey’s multiple-comparison test (*t* = 6.5 h). (*E*) Confocal image time series showing merging behavior (yellow arrowhead) of multiple individually developing interkingdom assemblages on the tooth-mimetic surface. Green, *S. mutans*; cyan, *C. albicans*. (Scale bar, 100 μm.)

Given that *C. albicans* and *S. mutans* can coassemble in saliva, followed by attachment to surfaces as a structured group, we investigated how the surface-attached cells residing in this biostructure grow spatiotemporally into biofilms. To investigate the growth dynamics of the sHA surface-attached assemblages, we employed a flow-cell microfluidic biofilm culture system that mimics saliva flow coupled with time-lapse confocal imaging and computational analyses (22) (Fig. 3*A*). We tracked each surface colonizer individually and calculated the dynamic change of its biovolume across the surface. These experiments showed that the interkingdom assemblage developed into larger biofilms compared to monospecies aggregates, eventually growing to cover the surface (Fig. 3*B*). Interestingly, when analyzing the biovolume of each species within the interkingdom assemblage, we found that the biovolume of *S. mutans* increased more rapidly than the biovolume of *C. albicans* (Fig. 3 *C*, *Inset*). We then compared the *S. mutans* biovolume growth dynamics within interkingdom assemblages to that of aggregated *S. mutans* alone or in assemblages treated with fungicide (using a high concentration of nystatin, selectively killing *C. albicans*). These experiments showed that the biovolume of *S. mutans* in the assemblages increased faster than *S. mutans* alone (Fig. 3*C*). Similarly, *C. albicans* inactivation with nystatin reduced the bacterial growth benefit from intact assemblages (Fig. 3*B*; quantification in Fig. 3*C*). Measurements of the surface coverage also show that the interkingdom assemblages spread much faster than *S. mutans* alone, or assemblages treated with the fungicide nystatin (Fig. 3*D*).

We also monitored the spatiotemporal growth dynamics of surface-attached aggregated *S. mutans* and interkingdom assemblages (Fig. 3*E*). When tracking two spatially distant assemblages, we found that they grew and expanded toward each other and eventually merged to create a new superstructure (Fig. 3 *E*, *Lower*). In contrast, individual *S. mutans* aggregates grow separately without merging events across the same time span (Fig. 3 *E*, *Upper*). As a result, the surface coverage occurred more rapidly by the interkingdom assemblage, compared to aggregated *S. mutans* or fungicide-treated assemblage (Fig. 3*D*).

### Assemblage Mobility, Bacterial Hitchhiking on Migrating Fungi, and Enhanced Surface Spreading.

**Fig. 4.**
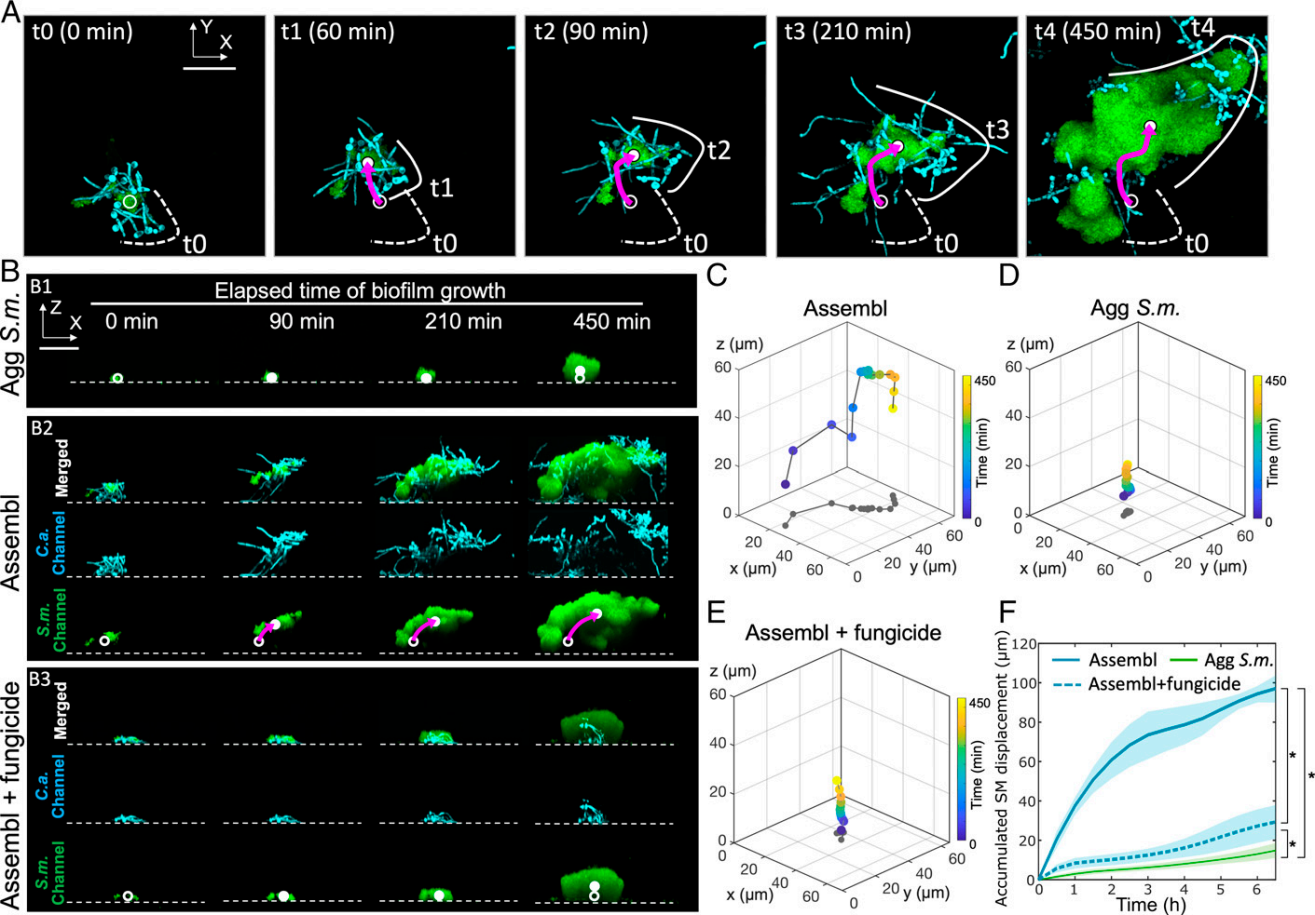
Dynamics of interkingdom group-level surface mobility and spreading. (*A*) Surface mobility of the colonized interkingdom assemblage during biofilm initiation. Solid line, leading edge of the interkingdom assemblage at different timepoints; dotted line, leading edge at initial position; solid dot, bacterial biovolume centroid at different timepoints; hollow dot, bacterial biovolume centroid at initial position; purple arrow, path of the bacterial biovolume centroid over time. Green, *S. mutans*; cyan, *C. albicans*. (Scale bar, 50 μm.) (*B*) Side-views (orthogonal projections) of selected time-frames obtained by time-lapse confocal microscopy for *B1*) aggregated *S. mutans* (Agg *S.m.*); *B2*: interkingdom assemblages of *S. mutans* and *C. albicans* (Assembl); *B3*: fungicide-treated assemblages (Assembl+fungicide). See Movies S1–S3 for *B1*–*B3*. Green, *S. mutans*; cyan, *C. albicans*. Solid dot, bacterial biovolume centroid at different timepoints; hollow dot, bacterial biovolume centroid at the initial position. (Scale bar, 50 μm.) (*C*–*E*) Four dimensional time-resolved trajectories of the bacterial biovolume centroids within surface-attached assemblage, aggregated *S. mutans,* and fungicide-treated assemblage. Color code: gray, projections of track onto *xy*-plane; other colors, time elapsed during the biofilm growth (0 to 450 min). (*F*) Accumulated *S. mutans* displacement (total path length) of the bacterial centroid relative to the initial position. Lines correspond to mean, shaded region to SD of *n* = 4 independent replicates. *P < 0.05 by one-way analysis of variance with Tukey’s multiple-comparison test (*t* = 6.5 h).

Next, we tracked the surface-attached interkingdom assemblages on surfaces and found a unique migratory behavior. In this migration process, the interkingdom assemblage deformed, and the “leading edge” (Fig. 4*A*, solid lines) moved significantly as time elapsed. In addition to the movement of the leading edge, the centroid of the *S. mutans* biovolume also moved across the surface (Fig. 4*A*, hollow dots indicate the centroid at the initial time point *t*0 and filled dots indicates the centroid at selected time points), suggesting a surface mobility of the assemblage. During the growth process, which coincides with the migration process, the interkingdom assemblage developed a changing directionality along the moving direction (Fig. 4*A*, purple arrow).

Since *S. mutans* cells are attached to *C. albicans* cells within the assemblage, and the killing of fungal cells by nystatin disrupted the surface-spreading of the bacteria within the assemblage (Fig. 3*D*), we hypothesized that the bacterial mobility across the surface by the interkingdom assemblages was driven by fungal growth. Using high-resolution time-lapse confocal microscopy and computational quantification of the mobility, we tested this hypothesis, and more generally explored how *S. mutans* and *C. albicans* codeveloped from the initial surface-bound assemblages into large biofilms. The fungal and bacterial behaviors within the surface-attached assemblages were analyzed individually from the four-dimensional (*x*, *y*, *z*, time) confocal datasets (orthogonal time-frames are shown in Fig. 4*B* and Movies S1–S3).

Surprisingly, the images showed that bacterial clusters were lifted away from the surface and transported laterally (purple arrows in Fig. 4 **B*2* and Movie S2) while continuously growing along with fungi, thus hitchhiking on the elongating hyphae. In contrast, aggregated *S. mutans* alone remained in their initial positions on the surface during the growth (Fig. 4 *B1* and Movie S1). Three-dimensional tracks of the centroid of the *S. mutans* biovolume within the assemblage show that the bacteria displayed lateral and vertical movement (Fig. 4*C*), whereas the monospecies *S. mutans* aggregates have very short tracks (Fig. 4*D*). Given that growing hyphae can generate mechanical forces at the point of contact (23), we investigated whether the mobility was resulting from the fungal hyphal growth. We specifically deactivated fungal growth in the assemblage using nystatin and examined the bacterial mobility behavior (Fig. 4 **B*3* and Movie S3). Spatial tracking analyses revealed a lack of filamentation and complete loss of lateral mobility within the interkingdom assemblages exposed to the fungicide nystatin (Fig. 4 *B*, 3 and *E*), similar to that of monospecies bacterial growth (Fig. 4 **B*1* and *D*), suggesting that hyphal formation was required for bacterial hitchhiking mobility. We calculated the accumulated path lengths of the biostructures relative to their initial positions (*t*0). The resulting displacement curves showed that the interkingdom assemblage moved at remarkably high velocity (up to 40 μm/h) during the first 3 h, then slowed down, and reached a total path length of ∼100 µm after 6 h (Fig. 4*F*). In contrast, centroids of aggregated *S. mutans* alone (or assemblages treated with nystatin to kill *C. albicans*) remained mostly stationary (<3 μm/h) with minimal displacement during growth (Fig. 4*F*). The differences in track length between *S. mutans* alone, and the assemblages illustrate that the larger mobility of the assemblage is not due to *S. mutans* growth.

**Fig. 5.**
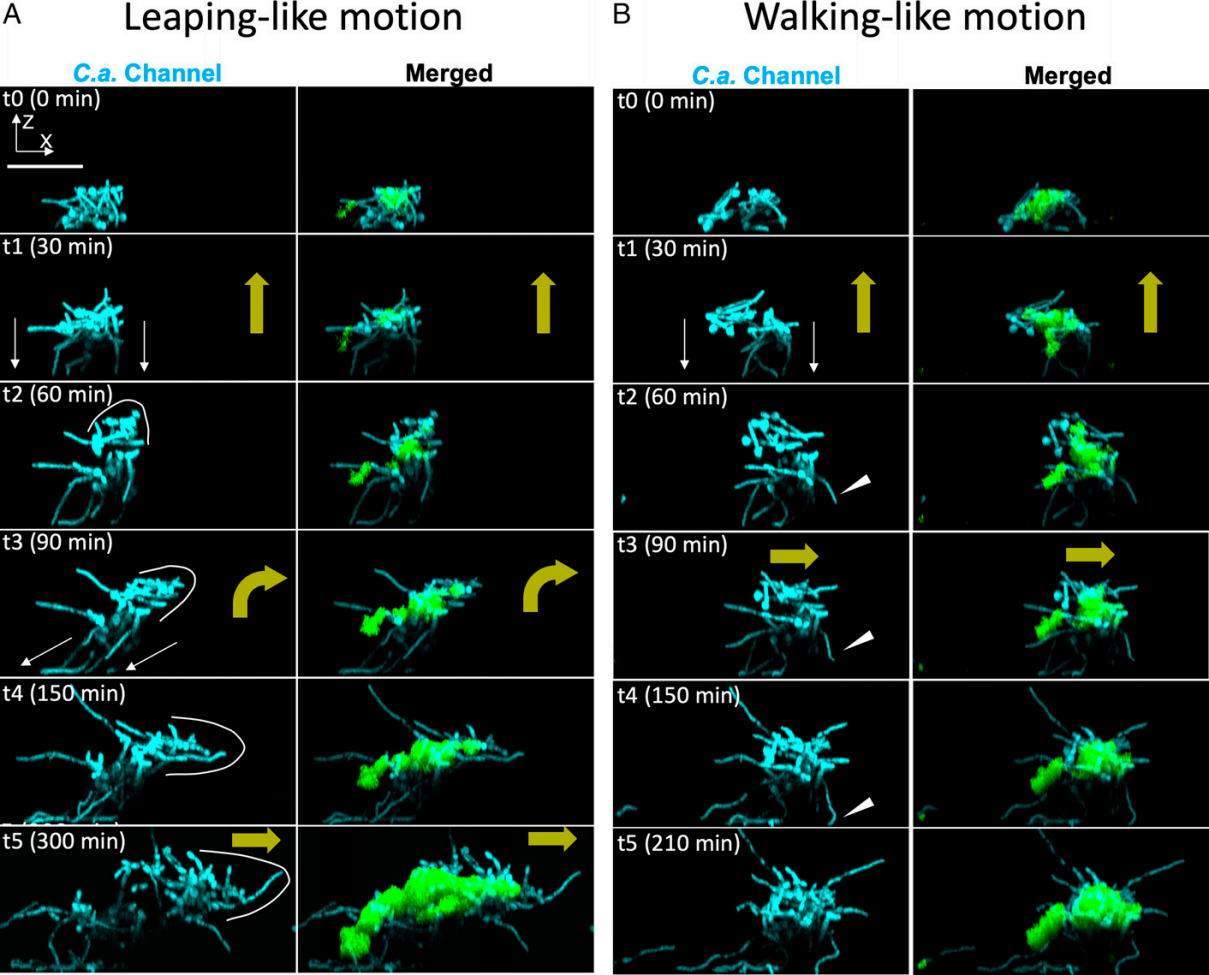
Assemblage mobility, bacterial hitchhiking, and spatial growth dynamics. Confocal image time series (*xz*-projections) for growing interkingdom assemblages. (*A*) Interkingdom assemblage displays a leaping-like motion. White lines indicate the leading edge of the growing assemblage. White thin arrows indicate the direction of fungal hyphal elongation toward the surface. (*B*) Walking-like motion. White arrowheads, newly formed fungal hyphae stablish new anchoring points on the surface. Green, *S. mutans*; cyan, *C. albicans*. Yellow thick arrows, moving directions of the assemblage. Abbreviation: *C.a.*, *C. albicans.* (Scale bar, 50 μm.)

We further assessed the hitchhiking motion of bacteria on fungi within surface-attached assemblage by analyzing the individual fluorescence channels (Fig. 5). The images revealed two interesting types of mobility: we found *C. albicans* filamentation from the fungal network and toward the surface (Fig. 5*A*, *t*1 in *C. albicans* fluorescence channel). Notably, the fungi moved upwards and then reoriented the leading edge (Fig. 5*A*, *t*2 to *t*4, white lines in *C. albicans* channel) to move laterally by an array of elongating hyphae pushing against the surface (Fig. 5*A*, *t*3, white arrows in *C. albicans* channel). The fungal network rapidly protruded to expand the leading edge of the biostructure laterally (Fig. 5*A*, *t*4 to *t*5), displaying behaviors that resemble, in anthropomorphic terms, a forward leaping-like motion (Movie S2). We also observed a “walking-like motion” (Fig. 5*B*) whereby, after moving up away from the surface, the assemblage established additional anchoring points by newly formed hyphae while moving across the surface (Fig. 5*B*, *t2* to* t4*, white arrowheads in *C. albicans* channel). Intriguingly, in both the leaping-like motion and walking-like motion, the bacterial clusters within the biostructure were lifted up above the surface and moved laterally across the surface as cargo attached onto the fungal cell (Fig. 5 *A* and *B*, merged channel). By hitchhiking, large bacterial clusters are transported by the hyphal-guided surface mobility while continuously growing, to spread away from the location of initial colonization.

Since the bacterial and fungal cells within the interkingdom assemblage are enmeshed by EPS α-glucans (Fig. 1*E*), we also assessed whether α-glucan degradation with glucanohydrolases (dextranase and mutanase) could impact the hitchhiking growth behavior. The data show that the presence of the enzymes during biofilm formation impaired bacterial attachment to the *Candida* hyphae (*SI Appendix*, Fig. S11). As a result, *S. mutans* cells were dislodged and could not hitchhike on the developing fungi despite continuous bacterial growth, suggesting that its ability to remain attached during the growth appears to be more important than the bacterial growth rate to keep pace with the hyphal movement.

Together, our data reveal a peculiar mechanism employed by the interkingdom assemblages in human saliva to promote surface colonization and coverage, enabled by bacterial hitchhiking on the fungal cell as hyphal forms grow and elongate on the surface. This remarkable structural organization and growth behavior enhance surface binding affinity while allowing mobility of surface-bound microbes to promote widespread colonization and biofilm formation.

### Enhanced Biofilm Virulence of Interkingdom Assemblage.

**Fig. 6.**
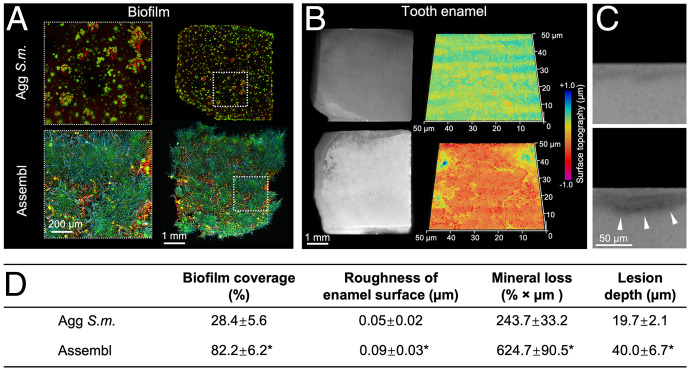
Interkingdom assemblage-mediated biofilm and tooth decay on human enamel. (*A*) On human enamel surfaces, biofilms derived from interkingdom assemblages (Assembl) or from aggregated *S. mutans* (Agg *S.m.*) were imaged using confocal microscopy. *Insets* with dotted lines show high-resolution view of magnified areas. Green, *S. mutans*; cyan, *C. albicans;* red, EPS matrix. (*B*) Multiscale analyses of the tooth-enamel underneath the biofilms. (*Left*) Macroscopic demineralized lesions (brighter, chalky areas) developed on the enamel surface when biofilms derived from interkingdom assemblages are present; (*Right*) corresponding surface topography analysis showing microcavities formed on the enamel surface. The surface topography is color-coded to visualize the microcavities. (*C*) Transverse microradiography of the human enamel surface underneath the biofilm derived from aggregated *S. mutans* (*Upper*) or biofilms derived from interkingdom assemblages (*Lower*). White arrowheads indicate area of enamel demineralization caused by biofilms derived from assemblages. (*D*) Quantitative analysis of biofilm surface coverage on human tooth-enamel, tooth surface topography, and tooth mineral analysis (mean ± SD of *n* = 4 independent replicates. *P < 0.05 by Student’s *t* test.

Given that the interkingdom assemblages displayed emergent behaviors, including enhanced growth, stronger stress tolerance, and migratory group-level mobility, we next investigated whether the acquired properties can enhance their disease-promoting function. We employed an ex vivo human tooth-enamel model that allows simultaneous analysis of the biofilm spatial structure and the extent of enamel decay (24). The biofilms were formed as described in *Materials and Methods* and the spatial distribution of the biofilm components and enamel surface characteristics were determined via a multilabeling approach, as well as surface topography analysis and transverse microradiography measurements (24). We found a highly cohesive and densely packed biofilm originating from the assemblages that covered the enamel surface (Fig. 6 *A*, *Lower*, and the parameter “biofilm coverage” in Fig. 6*D*). In contrast, the biofilm from aggregated *S. mutans* was sparsely distributed, leaving a substantial amount of uncolonized surface (Fig. 6 *A*, *Upper*, and “biofilm coverage” in Fig. 6*D*). The aggregated *C. albicans* alone was capable of binding and growing on enamel but the fungal cells were detached from the surface under fluid shear (*SI Appendix*, Fig. S12 *A* and *B*). The striking differences in both structural organization and surface coverage may cause differential damage on the human enamel tissue underneath.

To assess the enamel structural damage and mineral loss, we conducted surface analyses after removing the biofilms from the tooth enamel. Macroscopically, we found large areas of enamel demineralization associated with interkingdom assemblage-derived biofilm, which was characterized by chalky-like opaque surface under the stereoscope (Fig. 6 *B*, *Lower Left*), similar to those found clinically in severe childhood tooth decay. In contrast, only small areas of opaque demineralized areas were found on the enamel surface from aggregated *S. mutans* biofilms (Fig. 6 *B*, *Upper Left*). As expected, no significant enamel damage was found in biofilms formed by *C. albicans* alone (*SI Appendix*, Fig. S12*C*). These differences were confirmed with confocal topography imaging and transverse microradiography analysis. The enamel surfaces from interkingdom assemblages eroded severely and with widespread regions of surface damage compared to those from *S. mutans* aggregates, which showed milder enamel surface demineralization (Fig. 6 *B*, *Left*). Transverse microradiography analysis validated the extent of the decay, showing significantly higher mineral loss and deeper lesions inflicted by the biofilm originated from the assemblage (Fig. 6 *C* and *D*). Collectively, our findings reveal that the functionalities acquired by the interkingdom assemblages provide mutually beneficial symbiosis, enhancing surface colonization, survival, and spatial growth to potentiate biofilm virulence, causing more extensive and severe tooth decay, which cannot be achieved without coassembly.

## Discussion

**Fig. 7.**
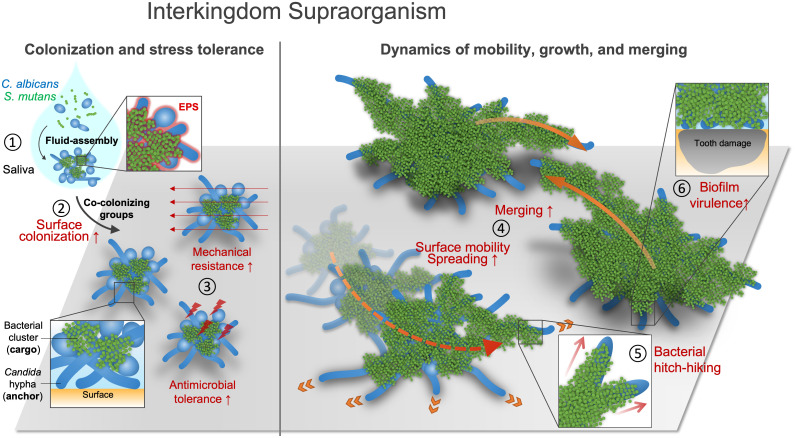
Interkingdom assemblages in human saliva behave like supraorganisms with new functionalities and disease-promoting activity. 1) *C. albicans* and *S. mutans* coassemble into structured cell groups in human saliva, which are remarkably similar to the native interkingdom aggregates found in intact saliva from diseased patients. 2) Bacteria and fungi collectively colonize the surface as a structured cell group with enhanced binding affinity. 3) The assemblage displays enhanced tolerance to shear stress and antimicrobials. 4) The assemblages behave as single units that grow faster than single-species aggregates, spreading three-dimensionally and merging with each other, resulting in high surface coverage. 5) The interkingdom assemblages display a novel mode of migratory group-level mobility with forward motions and a hitchhiking growth mechanism during biofilm initiation that allows nonmotile bacteria to relocate after surface colonization, which promotes biofilm spatial surface spreading. 6) The interkingdom assemblages cause extensive and severe damage of the tooth-enamel surface.

Microbial surface colonization is a critical first step for the biofilm formation, which requires microorganisms to tackle a range of environmental stresses to ensure surface binding, survival, and growth for successful community establishment. Our findings reveal an interkingdom bacterial–fungal assemblage in human saliva that behaves as a single organismal entity with emergent functions and new spatial growth mechanisms. The acquired functionalities of the interkingdom assemblage enhance the fitness of the microbes for efficient surface colonization and promote fast biofilm spreading on the tooth surface. We demonstrated five key features of the interkingdom assemblages, as summarized in Fig. 7. 1) *C. albicans* and *S. mutans* in human saliva form a structured assemblage comprised of bacterial clusters attached to a network of fungal cells and exoglucan matrix, which enables collective fungal-bacterial colonization as a preformed unit with enhanced surface binding affinity. 2) Upon colonization, the assemblages display emergent functions such as enhanced tolerance against shear stress and antimicrobials compared to either bacteria or fungi alone, promoting collective surface retention and survival. 3) The assemblages proliferate in 3D with a higher growth rate than single-species aggregates, rapidly merging with each other to form larger biofilms. 4) We find an unexpected group-level mobility, which allows collective migration of bacterial clusters attached to fungal cells with high speed and covering large distances on the surface, promoting biofilm spatial spreading. 5) The dynamic fungal–bacterial interactions lead to biofilm superstructures that cause extensive and more severe damage of the tooth-enamel surface. These findings suggest that taxonomically distinct microorganisms can organize into biostructures that display supraorganism-like properties, which refers to interacting individuals that behave in concert as a single unit with enhanced functions in analogy to a complex higher organism (25, 26).

The direct visualization of biofilm growth dynamics led to the discovery of a coordinated spatial mobility with peculiar leaping-like or walking-like motion. Bacterial cell clusters can hitchhike on this mobile unit while continuously growing, which promotes fast spreading across the surface. This collective multicellular migratory mode opens intriguing possibilities. This could be a stochastic mobility mechanism utilized by the interkingdom colonizers to boost range expansion nearby or possibly a navigation strategy to a desired direction or location. In nature, motile bacteria have developed a number of motility mechanisms to move around, exploiting environment and resources. Their locomotion depends on the use of different appendages (e.g., flagella, pili, or specialized motility apparatus) that allow different types of movement, including swimming, swarming, twitching, and gliding (27, 28). However, *S. mutans* is a nonmotile bacterium, whereas *C. albicans* is devoid of motility, although hyphal cells can elongate and penetrate soft tissues during mucosal infection (29, 30). The exact mechanisms for this migratory group-level motility behavior are unknown. Recent studies suggests that a single growing *Candida* hypha tip can generate sufficient forces driven by intracellular turgor pressure to penetrate host cell membrane or junctions (23, 31). It is possible that such forces are multiplied when the elongating pillar-like hyphae are in collision with the much stiffer tooth-mimetic apatitic surface, lifting the interkingdom assemblage while propelling attached bacterial clusters across the surface. Conversely, bacterial-derived EPS contribute to this process by providing stress tolerance and structural integrity that enable coordinated behaviors of bacteria and fungi as a cell group. Such group-level mobility on a surface presents a distinctive mechanism for community expansion of nonmotile bacteria, as no similar mechanisms have been reported previously, to the best of our knowledge. In addition, these mobile communities eventually merge with each other to enhance biofilm coverage across the tooth surface, causing more extensive and severe dental decay. Our work thus suggests a unique mechanism of microbial migration and biofilm spatial expansion with disease implications.

Bacteria and fungi can coadhere through different binding mechanisms, particularly between streptococci and *Candida* (as reviewed in refs. 32 and 33), raising the question of which components are acting in concert to build-up the interkingdom assemblage. We found that a triad of 1) fungal filamentation and 2) its cell-wall adhesins, particularly the agglutinin-like sequence (Als) family (34), as well as 3) bacterial-derived EPS α-glucans (13) are critical for the bacterial–fungal coassembly in saliva and colonization as structured assemblages. Impairment of each of these factors impacts both the emergent properties and surface group-mobility, which indicates a highly intertwined, codependent mechanism. Notably, the *ΔΔals1/ΔΔals3* deletion caused a severe reduction of *C. albicans*–*S. mutans* coassembly in saliva and surface cocolonization. *C. albicans* can physically interact with mitis group streptococci through Als adhesins (32, 33), which may also mediate *C. albicans–S. mutans* assemblage via direct binding to *S. mutans* through cell-surface proteins or EPS-glucans, as Als are capable of inducing coaggregation with other bacteria (35). In addition, Als can bind to salivary proteins (36) known to interact with the *S. mutans* cell surface (37), which could indirectly promote coadhesion. How these interactions occur dynamically and whether there are other governing factors (e.g., host salivary components or other dietary factors) require further elucidation. In addition, microbial species often develop complex chemical interactions within the shared habitat, including metabolic exchange, cross-feeding, and cell–cell communication (38). A complex cross-feeding has been observed between *C. albicans* and *S. mutans* in established biofilms involving sugar cometabolism that promotes fungal growth and bacterial Gtf production (39, 40), which may impact their structural organization, growth, and pathogenic traits during biofilm development.

Although the etiology of tooth decay is multifactorial (41), the disease is driven by dietary sugars, among which sucrose is a key determinant (9). Recent studies show that high sucrose intake is associated with increased *S. mutans* counts in saliva and increased risk of childhood caries (42), while the sugar consumption often outranks other disease-associated factors (43). Sucrose is essential for virulence because, in addition to being fermentable, it serves as substrate for EPS–glucan synthesis by *S. mutans*-derived Gtfs, promoting interkingdom coadhesion, colonization, and biofilm formation. Notably, *C. albicans* can increase EPS matrix accumulation by Gtf induction via quorum-sensing molecules (39), creating diffusion-limiting barriers that facilitate acid retention at the biofilm–apatite interface (13). Conversely, *S. mutans* convert sucrose to glucose, which is more readily metabolized by *C. albicans* to enhance fungal growth and acid production (16, 17). Hence, the cometabolism in conjunction with emergent properties promotes acidogenic biofilm spreading with higher biomass and surface coverage, contributing to the increased tooth decay caused by interkingdom assemblages. How metabolic exchanges are orchestrated in the presence of other oral pathogens or commensals and whether polymicrobial interactions (13) alter the growth dynamics of the interkingdom assemblage remain unknown. Future studies using ex vivo and in vivo polymicrobial models are needed to investigate the spatial–temporal dynamics during interkingdom biofilm development in complex communities.

In summary, an interkingdom symbiotic assemblage is found in human saliva that behaves like a supraorganism with emergent functionalities to enhance surface colonization, survival, and microbial growth dynamics. We discovered an intriguing multidirectional group-level mobility that allows migration of nonmotile bacterial clusters coordinated by EPS and fungal filamentation. These interkingdom assemblages rapidly spread on surface as “mobile growth nuclei” that merge with each other to expand tridimensionally, leading to large biofilm superstructures, which results in disease-causing activities. These observations could be of clinical importance to provide insights into the onset of severe childhood caries characterized by rapid and aggressive decay of the tooth enamel. This codevelopment could potentially be therapeutically exploited, for example, by the local applications of antifungal agents or EPS-degrading enzymes to block the biofilm spreading. Such dynamic, coordinated interkingdom unity behavior may also occur in other biofilm-forming communities causing human diseases and biofouling in environmental settings.

## Materials and Methods

### Sample Collection and Ethics Statement.

We collected dental plaque and saliva samples from healthy (caries-free) children and children with severe early-childhood caries following established protocols (44). Briefly, dental plaque on all the smooth-tooth surfaces from healthy (*n* = 14) and diseased children (*n* = 30) was collected using sterilized periodontal scalers and immediately transported to the laboratory on ice. Plaque samples were sonicated to disperse the cells without affecting cell viability, and then serially diluted and plated onto CHROMagar and Mitis Salivarius with Bacitracin, which selectively detect *C. albicans* and *S. mutans*. Colony forming units (CFU) of *C. albicans* and *S. mutans* in each sample were determined. For imaging analyses, unstimulated saliva from children was collected using sterile saliva ejectors (44) and transferred immediately to the laboratory on ice. For experimental models requiring saliva-coated apatitic surfaces (tooth-enamel surrogate) or saliva-based culture medium, whole saliva was pooled and filter‐sterilized. Human tooth-enamel specimens for the ex vivo tooth-enamel biofilm model were prepared from de-identified extracted human teeth. All protocols were approved by the Research Subject Review Board of the University of Rochester (RSRB #1248), the Institutional Review Board of the University of Pennsylvania (IRB #824243), and the Institutional Review Board of Indiana University (IRB #NSO911-07). Additional details are in *SI Appendix*.

### Multiscale Analyses of Native-State Microbial Biostructures in Saliva.

For confocal imaging, bacteria in saliva samples were stained with 0.1 μM Syto9 and fungi were labeled with 40 μg/mL Concanavalin A-tetramethylrhodamine (11). To visualize the streptococcal EPS α-glucans on the bacterial and fungal cell surfaces, we incubated the saliva sample with Alexa Fluor 647-labeled dextran conjugate in the presence of 1% sucrose (11). The Gtf activity in saliva was analyzed using scintillation counting (45). FISH of the native-state microbial biostructures in saliva was performed using species-specific oligonucleotide probes (46, 47) (*SI Appendix* for details).

### Microorganisms and Growth Conditions Used in Experimental Analyses.

*S. mutans* UA159 and *C. albicans* SN250, which are associated with severe childhood caries (10), were used to investigate the interkingdom interaction in saliva and in the biofilm. Microorganisms were grown in ultrafiltered (10-kDa cutoff) buffered tryptone-yeast extract broth (UFTYE; 2.5% tryptone and 1.5% yeast extract) to exponential phase (37 °C, 5% CO_2_). For flow-cell microfluidic live-imaging analysis, a fluorescent protein (tdTomato)-tagged *C. albicans* SN250 strain was used. *C. albicans* homozygous knockout strains, including *ΔΔefg1*, *ΔΔals1/ΔΔals3*, *ΔΔhwp2*, *ΔΔhyr1*, *ΔΔeap1*, and *S. mutans ΔgtfBC* were used to investigate the roles of hyphal formation, adhesins, and streptococcal glucan formation in the interkingdom assemblage and colonization. Concanavalin A-tetramethylrhodamine was used to label *C. albicans* mutant strains (11). *S. gordonii* DL1, an early-colonizer oral bacterium (13), was used in the experiments to assess mechanical resistance and antimicrobial tolerance of interkingdom assemblages.

### Experimental Model for Studying Interkingdom Assemblages and Surface Colonization in Saliva.

A fluid-to-surface colonization model was developed to investigate the dynamics of interkingdom assemblage and surface colonization from saliva to sHA based on established protocols (48). Hydroxyapatite disks (2.7 ± 0.2 cm^2^) were preincubated in filter-sterilized saliva to form the salivary pellicle. The pellicle is formed by selective adsorption of salivary proteins and other biomolecules, which mimics the biochemical properties of in vivo tooth-enamel surfaces modulating the colonization of oral microbes (13). *C. albicans* (10^5^ CFU/mL) and *S. mutans* (10^7^ CFU/mL) were incubated (60 min, 37 °C) in filter‐sterilized saliva supplemented with 1% sucrose. Then, vertically placed sHA disks were immersed in the saliva containing *C. albicans* and/or *S. mutans* to allow microbial binding (60 min, 37 °C). To visualize the attached cells, the disk was stained with Syto9 and Concanavalin A-tetramethylrhodamine. A separate set of disks was used for microbiological analysis. To assess roles of *S. mutans*-derived EPS during the colonization, glucanohydrolases (dextranase and mutanase) that specifically digest the α-glucans produced by *S. mutans* GtfB and GtfC (20) were added in the saliva. Additional details are in *SI Appendix*.

### In Situ Mechanical Resistance and Antimicrobial Tolerance of Surface-Attached Biostructures.

The mechanical stability of surface-attached microbial biostructures was investigated by applications of fluid shear stress and assessment of surface detachment via a flow-cell microfluidic imaging device (BioSurface Technologies). The disk with the surface-attached, prestained biostructures was mounted in the device, which was connected to a peristaltic pump (Cole-Parmer) and coupled with a confocal microscope. Disks were subjected to a controllable flow (from 0.1 to 200 mL/min). A computational fluid dynamics module of COMSOL (V5.2) was used to estimate the fluidic wall shear stress at the surface. This setup allows applying varying wall shear stress (from 0.001 to 20 Pa) and assessing the detachment of assemblages in real time. Each fluid sheer stress was applied for 60 s, then paused during image acquisition. The multichannel image was subject to computational analysis to determine the biovolume. For interkingdom assemblages, the remaining biovolume was calculated as the total biovolume of *S. mutans* and *C. albicans* that remained on the surface. Relative mechanical resistance is defined as the ratio of remaining biovolume to the original biovolume. To investigate antimicrobial tolerance, we developed an in situ cell viability staining/imaging technique allowing real-time visualization of killed microbial cells (49). Briefly, disks with surface-attached, prestained microbes were immersed in 1 µM Toto-3, a cell-impermeable dimeric cyanine acid dye as a real-time cell-death indicator for both bacteria and fungi (49). Chlorhexidine (100 µg/mL), a broad-spectrum antimicrobial that kills both bacteria and fungi (50), and nystatin (250 µg/mL), an oral fungicide without antibacterial effects (21), were used. We also determined the CFU of *C. albicans* and *S. mutans* after 5-min chlorhexidine or 20-min nystatin treatment. Pretreatment with dextranase and mutanase (20) was also conducted to assess whether EPS α-glucan degradation could affect the mechanical and antimicrobial tolerance.

### Dynamics of Biofilm Initiation from Interkingdom Assemblage.

Biofilm growth dynamics and spatial organization of individual aggregates were tracked by time-lapsed confocal imaging coupled with flow-cell microfluidics based on an established protocol (22). The sHA disk with the initial colonizing community were prestained with Syto9 to label *S. mutans* cells. We used the fluorescent (tdTomato) *C. albicans* SN250 strain for tracking the fungal growth. Then, the disk was aseptically transferred into the microfluidics device. UFTYE with 25% saliva and 1% sucrose was provided (100 μL/min) using a peristaltic pump. The medium was supplemented with 250 nM Syto9 to allow continuous bacterial cell labeling in the growing biofilm (48). Bacterial EPS α-glucans were labeled via 1 μM Alexa Fluor 647-dextran during biofilm growth (11). Time-lapsed confocal imaging (*z*-stacks of 0.31-μm pixel size; 1-μm *z*-step) was performed every 30 min at 37 °C using a 40× water-immersion objective (numerical aperture = 1.2) on the Zeiss LSM800 microscope. Additional details are in *SI Appendix*.

### Computational Structural Analysis and Tracking.

General computational image processing and quantitative analysis were performed using BiofilmQ software (https://drescherlab.org/data/biofilmQ) (15). Structural analysis and tracking were performed using BiofilmQ in combination with customized MATLAB scripts (15). Briefly, for spatial measurements, segmented channels were merged and the object parameter “RelativeAbundance_chx” was used to measure the biovolume abundance for each channel within small cubic volumes. To determine the distribution of *C. albicans*, *S. mutans*, or EPS across the height of the microbial structure, the cubes were assigned to different horizontal sections (thickness = 2.5 µm) parallel to the surface, based on the *z*-coordinate. The mean relative abundance in each section was calculated and the height was normalized by the *z*-coordinate of the microbial structure’s center-of-mass (centroid). To measure the biofilm volume over time, the global parameter “Biofilm_Volume” was used. Each growth curve was normalized by the mean of “Biofilm_Volume” for timepoints *t*_1.5h_, *t*_2h_, and *t*_2.5h_, which have improved signal-to-noise ratio than earlier timepoints for optimized normalization. For the group-level mobility tracking, we determined the centroid of the bacterial clusters over time to generate time-resolved 3D trajectories. The accumulated *S. mutans* displacement of the bacterial centroid relative to the initial position was calculated using spatial coordinates at each timepoint. We also analyzed the dynamics of biofilm surface coverage, which is defined as the sum of all segmented pixels in *z*-projections, normalized by the mean value of *t*_1.5h_, *t*_2h_, and *t*_2.5h_. Additional details are in *SI Appendix*.

### Ex Vivo Human Tooth-Enamel Biofilm Model.

To investigate the disease-promoting functions, we employed an ex vivo human tooth-enamel model that allows simultaneous analysis of the biofilm spatial structure and the extent of enamel decay underneath (24). Briefly, interkingdom assemblage, aggregated *S. mutans*, or aggregated *C. albicans* were allowed to bind and form biofilms onto sterilized human-enamel specimens following the same fluid-to-surface colonization protocol as stated above. The enamel specimens were incubated in filter‐sterilized saliva with 1% sucrose for 67 h. The biofilm structural organization on the tooth-enamel surface was assessed via a multilabeling approach as detailed in *SI Appendix*. The biofilms were imaged using a 20× water-immersion objective (numerical aperture = 1.0) on the Zeiss LSM800 system. Amira software (v5.4.1) was used to generate renderings of the biofilm 3D architecture.

### Enamel Surface Analyses.

To assess the enamel structural damage and mineral loss, we conducted multiscale surface analyses after removing the biofilms from the tooth enamel (24, 39). In brief, after biofilm imaging, the biomass was removed using enzymatic treatment (dextranase and mutanase) followed by water-bath sonication, which was optimized for biofilm removal without causing artificial surface damage (24). Macroscopically, the demineralized areas on tooth-enamel surfaces (similar to those found clinically in severe childhood tooth decay) were visualized using stereomicroscopy (Zeiss AxioZoom v16). Then, the surface topography and roughness of the tooth-enamel surface were assessed by nondestructive confocal-topography using a 50× (numerical aperture = 0.95) objective on the Zeiss LSM800 microscope and ConfoMap software (24). Next, the tooth-enamel specimens were mounted on acrylic rods and sectioned (100 ± 20 μm thickness) with a hard-tissue microtome (Silverstone-Taylor Hard Tissue Microtome, Series 1000 Deluxe) for transverse microradiography. The sections were placed in the TMR-D system and X-rayed (45 kV, 45 mA) at a fixed distance for 12 s. An aluminum step wedge was X-rayed under identical conditions. The digital images were analyzed using TMR software (v3.0.0.18), with sound enamel defined at 87% mineral volume (51).

### Statistical Analysis.

Experimental data were presented with mean ± SD. Data were subjected to Student’s *t* test or ANOVA with post hoc tests (Dunnett’s or Tukey’s test) for a multiple comparison. Nonnormally distributed data from clinical samples are described as median ± interquartile ranges and were analyzed by Mann–Whitney *U* test. Differences between groups are considered statistically significant when *P* < 0.05.

## Supplementary Material

Supplementary File

Supplementary File

Supplementary File

Supplementary File

## Data Availability

The customized MATLAB codes (BiofilmQ) used for the computational image analysis can be found at GitHub [https://github.com/knutdrescher/interkingdom-assemblage-quantification (52)]. All other study data are included in the main text and supporting information.
