# Supplementary material for: Interkingdom assemblages in human saliva display group-level surface mobility and disease-promoting emergent functions
Source: Proc Natl Acad Sci U S A. 2022 Oct 3;119(41):e2209699119. doi: 10.1073/pnas.2209699119 (PMC9565521; doi:10.1073/pnas.2209699119)
Supplement: Supplementary File [file pnas.2209699119.sapp.pdf]

PNAS

Supplementary Information for

Interkingdom assemblages in human saliva display group-level surface mobility and disease-promoting emergent functions

Zhi Ren, Hannah Jeckel, Aurea Simon-Soro, Zhenting Xiang, Yuan Liu, Indira M Cavalcanti, Jin Xiao, Nyi-Nyi Tin, Anderson Hara, Knut Drescher, and Hyun Koo

Corresponding authors:

Knut Drescher (knut.drescher@unibas.ch)

Hyun Koo (koohy@upenn.edu)

This Supplementary Information includes:

SI Methods

Figures S1 to S12

Legends for Movies S1 to S3

Other supplementary materials for this manuscript include the following:

Movies S1 to S3

SI Methods

Sample collection and ethics statement. We collected dental plaque and saliva samples from healthy (caries-free) children and diseased children diagnosed with severe early childhood caries, as defined by the 2014 Reference Manual of the American Academy of Pediatric Dentistry, following established protocols (1). Briefly, dental plaque on all the smooth tooth surfaces from healthy (n=14) and diseased children (n=30) was collected using a sterilized periodontal scaler, with written informed consent signed by their legal guardians. Samples were transferred into 1 mL phosphate-buffered saline (PBS) in a sterilized Eppendorf tube and were immediately transported to the laboratory on ice. Plaque samples were gently vortexed and sonicated (three 10-s pulses with 30-s intervals at 7 W) to disperse the cells without affecting their viability, and then serially diluted and plated within 2 hours onto BBL CHROMaga *Candida* Medium (BD, Sparks, MD, USA) and Mitis Salivarius with Bacitracin Medium, which selectively detect *C. albicans* and *S. mutans*, respectively. After incubation (37 °C for 48 hours), the total numbers of *C. albicans* and *S. mutans* cells in each plaque sample were determined by colony forming units (CFU). For imaging analyses, unstimulated saliva from healthy and disease children was collected using a sterile disposable saliva ejector following established clinical protocols (1). Approximately 0.5 mL saliva was collected from each subject. The unprocessed sample was stored on ice and transferred immediately to the laboratory for imaging analyses. For experimental models that require saliva-coated surfaces (as tooth-enamel surrogate) or saliva-based culture medium, whole saliva was collected immediately before each experiment from at least two healthy donors and then pooled into a single source before filter-sterilization (0.22-μm pore size, low protein-binding membrane). Human tooth enamel specimen (4 mm × 4 mm) for the *ex vivo* tooth-enamel biofilm model were prepared from de-identified extracted human teeth previously collected at the Oral Health Research Institute, Indiana University School of Dentistry. All protocols were reviewed and approved by the Research Subject Review Board at the University of Rochester (RSRB #1248), the Institutional Review Board of University of Pennsylvania (IRB #824243), and the Institutional Review Board of Indiana University (IRB #NSO911-07).

Multiscale analyses of native-state microbial biostructures in saliva. For confocal imaging, bacterial cells in saliva samples were stained with 0.1 μM Syto9 (Molecular Probes) and fungal cells were labelled with 40 μg/mL Concanavalin A-tetramethylrhodamine conjugate (Molecular Probes) (2). To visualize the streptococcal EPS α-glucans on the bacterial and fungal cell surfaces, we incubated the saliva sample with Alexa Fluor 647-labeled dextran conjugate (Molecular Probes) for 4 h in the presence of 1% sucrose, which is a highly specific labelling method for streptococcal glucosyltransferase (Gtf)-derived α-glucans (2). The Gtf activity in the saliva was analyzed using scintillation counting as described previously (3) by measuring the incorporation of [^{14}C] glucose from radiolabeled sucrose substrate (New England Nuclear Research Products, Boston, MA) into glucan products for 4 h at 37 °C. One unit (U) of Gtf enzyme is defined as the amount of enzyme that can incorporates 1 μmol glucose into glucan over a 4-h reaction. Fluorescence *in situ* hybridization (FISH) analysis of the native-state microbial biostructures in saliva was performed as detailed previously (4, 5), using species-specific FISH oligonucleotide probes including: 5′-ACTCCAGACTTTCCTGAC-3′ (SMU587) with Cyanine-5 (Cy5) for *S. mutans* and 5′-GCCAAGGCTTATACTCGCT-3′ (CAAL) with fluorescein for *C. albicans*. Immediately before imaging, the sample was gently pipetted onto pre-solidified 1% agarose on a glass slide followed by a coverslip to immobilize the microbes while preserving their native structure (6). Super-resolution confocal imaging was performed using a Zeiss LSM800 microscope with Airyscan and a 40× water immersion objective with numerical aperture (NA) of 1.2.

Microorganisms and growth conditions used in experimental analyses. *S. mutans* UA159 and *C. albicans* SN250, which are associated with severe childhood caries (7), were used to investigate the interkingdom interaction in saliva and in the biofilm. The microorganisms were grown in ultra-filtered (10-kDa molecular-mass cutoff membrane) buffered tryptone-yeast extract broth (UFTYE; 2.5% tryptone and 1.5% yeast extract) to exponential phase (37 °C, 5% CO_2). For flow-cell microfluidic live imaging analysis, a fluorescent protein (tdTomato) tagged *C. albicans* SN250 strain (*C. albicans* SN250 tdTomato) was used. *C. albicans* homozygous knockout strains, including *C. albicans ΔΔefg1*, *ΔΔals1/ΔΔals3*, *ΔΔhwp2*, *ΔΔhyr1*, *ΔΔeap1* and *S. mutans ΔgtfBC* were used to investigate the roles of fungal hyphal formation, adhesins and streptococcal glucan formation in the interkingdom assemblage and colonization. Given that the *C. albicans* mutant strains do not express a fluorescent tag, we used Concanavalin A-tetramethylrhodamine conjugate

(Molecular Probes) to fluorescently label the *C. albicans* cell wall in these experiments (2). *Streptococcus gordonii* DL1, an early-colonizer commensal oral bacterium (8), was used in the experiments to assess mechanical resistance and antimicrobial tolerance of interkingdom assemblages.

Experimental model for studying interkingdom assemblages and surface colonization in saliva. An *in-vitro* fluid-to-surface colonization model was developed to investigate the dynamics of interkingdom assemblage and surface colonization from saliva to saliva-coated hydroxyapatite (sHA), a tooth-mimetic surface, based on established protocols (6). Hydroxyapatite disks (2.7 ± 0.2 cm^2; Clarkson Chromatography Products) were pre-incubated in filter-sterilized human saliva (60 min at 37 °C) to form the salivary pellicle. The pellicle is formed by selective adsorption of salivary proteins, glycoproteins, and other biomolecules onto the surface, which mimics the biochemical properties of tooth enamel surfaces *in vivo* and modulates the initial microbial colonization of oral microbes (8). To recapitulate the interkingdom interaction, *C. albicans* (10^5 CFU/mL, yeast form) and/or *S. mutans* (10^7 CFU/mL) were incubated (60 min at 37 °C) in whole human saliva which was pooled from healthy donors, filter-sterilized (0.22-µm pore size, low protein-binding membrane), and supplemented with 1% sucrose. Then, sHA disks placed in a vertical position using custom-holders (to mimic the smooth surface of human teeth) were immersed in the saliva inoculum containing *C. albicans* and/or *S. mutans* to allow microbial binding (60 min at 37 °C). To visualize the bacterial and fungal cells attached on the surface, disks with the initial colonizing community (including interkingdom assemblages and single cells) were pre-stained with 0.1 µM Syto9 (for *S. mutans*) and Concanavalin A-tetramethylrhodamine (for *C. albicans*). Then, the disks were gently washed in 0.9% sodium chloride solution to remove loosely bound microorganisms before imaging. To determine the number of fungal and bacterial cells attached onto the surface, a separate set of disks was used for microbiological analysis. Fungal and bacterial cells were removed from the surface by sonication (three 10-s pulses with 30-s intervals at 7 W) to disperse the cell clusters without affecting their viability. Samples were serially diluted, plated, and the number of viable cells (CFU) for both *S. mutans* and *C. albicans* on each disk were determined. To assess the role of *S. mutans*-derived EPS α-glucan matrix during the colonization, glucanohydrolases (dextranase and mutanase) that specifically digest the α-glucans produced by *S. mutans* GtfB and GtfC (9) were added in the saliva.

***In-situ* mechanical resistance and antimicrobial tolerance of surface-attached biostructures.** The mechanical stability of surface-attached microbial biostructures was investigated by applications of fluid shear stress and assessment of surface detachment via a flow-cell microfluidic imaging device (BioSurface Technologies). The sHA disk with the pre-stained (Syto9 for *S. mutans* and Concanavalin A-tetramethylrhodamine for *C. albicans*), surface-attached biostructures was gently washed to remove loosely bound microbes and mounted in the flow-cell microfluidic device. The device was connected downstream to a digital peristaltic pump (Cole-Parmer) and was coupled with the Zeiss LSM800 confocal microscopy. Disks were subjected to a controllable flow (from 0.1 to 200 mL/min). The Computational Fluid Dynamics module of COMSOL Multiphysics (V5.2) was used to estimate the fluidic wall shear stress at the surface on which the biostructures were attached. This setup allows applying varying wall shear stress ranging from 0.001 to 20 Pa and assessing the detachment of the assemblages in real-time using confocal imaging. Each fluid sheer stress was applied for 60 s then paused during image acquisition. The multi-channel confocal image was subject to thresholding and the biovolume was determined. For interkingdom assemblages, the remaining biovolume was calculated as the total biovolume of *S. mutans* and *C. albicans* that remained on the surface after the flow. Relative mechanical resistance is defined as the ratio of remaining biovolume on the surface to the original biovolume. To investigate the tolerance of the biostructures to antimicrobials, we developed an *in-situ* cell viability staining and imaging technique allowing real-time visualization of killed microbial cells, based on modifications of our previous protocol (10). Briefly, disks with surface-attached microbes were pre-stained with Syto9 for *S. mutans* and with Concanavalin A-tetramethylrhodamine for *C. albicans*. The disks were immersed in 1 µM Toto-3 (Molecular Probes), a cell impermeable dimeric cyanine acid dye as a real-time cell death indicator for both bacteria and fungi (10). The time point denoted as "0 min" corresponds to the first confocal image acquisition before adding the antimicrobial agent. Chlorhexidine (100 µg/mL), a commonly used broad-spectrum antimicrobial used in dental clinics that can kill both bacteria and fungi (11) and nystatin (250 µg/mL), a clinically used oral fungicide that has no killing effect on bacteria (12) were used as model antimicrobial agents. We also

quantitatively assessed the antimicrobial resistance of fungal/bacterial cells in different biostructures by determining the residual viable cell number (CFU) of *C. albicans* and *S. mutans* after the treatment (5 min after chlorhexidine or 20 min after nystatin treatment). To test whether EPS α-glucan degradation could affect the mechanical and antimicrobial resistance, the disks with the attached biostructures were pre-treated with glucanohydrolases (dextranase and mutanase) that specifically break down the α-glucans produced by *S. mutans* (9) before the experiment.

Dynamics of biofilm initiation from interkingdom assemblage. Biofilm growth dynamics and spatial organization of individual aggregates were tracked by a time-lapsed confocal imaging system coupled with flow-cell microfluidics, based on our continuous flow-cell labelling and confocal imaging protocol (13). The HA disk with the initial colonizing community (including interkingdom assemblages and single cells) were pre-stained with 0.1 µM Syto9 to label *S. mutans* cells. We used the fluorescent *C. albicans* SN250 strain (*C. albicans* SN250 tdTomato) for tracking the fungal cells. Then, the disk was gently washed to remove loosely bound microbes and aseptically transferred into a flow-cell microfluidics device (FC310, BioSurface Technologies) for biofilm development analysis via time-lapsed confocal imaging. UFTYE medium supplemented with 25% saliva and 1% sucrose was continuously provided (100 µL/min) using a peristaltic pump to mimic the natural nutrient condition of dental plaque in the oral cavity. The medium was supplemented with 250 nM Syto9 to allow continuous bacterial cell labelling in the growing biofilm. The concentration was pre-determined to yield optimized bacterial cell labeling without negative effects on cell growth (6). Bacterial EPS glucan matrices were labeled via supplementing the culture medium with 1 µM Alexa Fluor 647 dextran conjugate (Molecular Probes) during biofilm growth. This labeling method is highly specific for *S. mutans*-derived α-glucans since the fluorescently-labeled dextrans serve as primers for streptococcal Gtfs and are directly incorporated into glucans during biofilm EPS synthesis (2). Time-lapsed confocal imaging (z-stacks of 0.31-µm pixel size and 1 µm z-step) was performed every 30 min at 37 °C using a 40× water-immersion objective (numerical aperture = 1.2) on the Zeiss LSM800 microscope with Airyscan functionality. The growing biofilm was sequentially scanned (488/640-nm lasers for Syto9/Alexa Fluor 647-dextran, then 561-nm laser for Concanavalin A-tetramethylrhodamine) and the emitted signal was collected using optimum emission wavelength filters. Image visualization was performed using ImageJ Fiji (https://imagej.net/Fiji).

General image processing. Computational image processing and quantitative analysis were performed following established protocols using BiofilmQ software (https://drescherlab.org/data/biofilmQ) (14), an image analysis toolbox optimized for biofilms. Briefly, multi-channel raw images (*S. mutans*, *C. albicans*, and EPS) were imported into BiofilmQ and the stage drift (for time-lapse imaging data) was corrected using the image alignment function. Individual microbial biostructures (interkingdom assemblage, aggregated *S. mutans*, and aggregates *C. albicans*) were cropped to generate designated datasets. After average filtering, each channel of the image was segmented using Otsu algorithm with 2 classes, multiplied with a sensitivity value of 0.35. The thresholding result was verified visually by users and further optimized when needed to ensure precise segmentation. After segmentation, a cube-based object declumping was performed, which dissected a larger biofilm volume into small cubic volumes. A cube side length of 10 pixels was used for biovolume or surface coverage measurements and a 4-pixel cube was used for spatial measurements. This function allows further analysis of biofilm properties inside the biofilm volume with spatial resolution because each cube has a unique spatial coordinate in 3D.

Computational structural analysis and tracking. Computational structural analysis and tracking were performed using BiofilmQ in combination with customized MATLAB scripts to generate specific plots (14). In brief, for spatial measurements, the three segmented (binarized) channels corresponding to *S. mutans*, *C. albicans*, and EPS were merged using BiofilmQ. The object parameter "RelativeAbundance_chx" was used to measure the biovolume abundance for each channel within each cube. To determine the distribution of each components (*C. albicans*, *S. mutans*, or EPS) over the height of the microbial structure (i.e., from the surface to the top of the structure), the cubes were assigned into horizontal sections (thickness = 2.5 µm) parallel to the surface at different heights, based on the z-coordinate of each cube. The mean relative abundance in each section was calculated and the height was normalized by the z-coordinate (vertical position) of the microbial structure's center-of-mass (or "centroid"). The resulting spatial distribution curves

were smoothed using MATLAB's built-in smooth function. To measure the biofilm volume over time, the global parameter "Biofilm_Volume" in BiofilmQ was used. To ensure a meaningful comparison, each growth curve was normalized by the initial biovolume, defined as the mean of the BiofilmQ parameter "Biofilm_Volume" of the timepoints $t_{1.5h}$, t_{2h}, and $t_{2.5h}$. These timepoints were determined to have improved signal-to-noise ratio than those earlier than 1.5 h, leading to optimized normalization with minimal systemic error. For the group-level bacterial mobility tracking, we determined the biovolume centroid of the bacterial clusters over time based on the segmentation performed in BiofilmQ. Time-resolved 3D trajectories were generated using the spatial coordinates of the bacterial biomass at each timepoint. The accumulated *S. mutans* displacement (total path length) of the bacterial centroid relative to the initial position was calculated. We also analyzed the dynamics of biofilm surface coverage, which was defined as the sum of all segmented pixels of the largest connected object in the z-projection on the entire surface, normalized by the mean value across $t_{1.5h}$, t_{2h}, and $t_{2.5h}$ (due to improved signal-to-noise ratio than the earlier timepoints). The Code Availability section describes how the customized MATLAB codes used for the computational image analysis can be obtained.

***Ex-vivo* human tooth-enamel biofilm model.** To investigate the disease-promoting functions of the interkingdom assemblage, we employed an *ex-vivo* human tooth-enamel model which allows simultaneous analysis of the biofilm spatial structure and the extent of enamel decay underneath (15). Briefly, interkingdom assemblage, aggregated *S. mutans*, or aggregates *C. albicans* were allowed to bind onto vertically-mounted sterilized human enamel specimens (4 mm × 4 mm) following the same protocol as detailed in *Experimental model for interkingdom assemblage and surface colonization in saliva*. The enamel specimens were gently washed in 0.9% sodium chloride solution to remove loosely bound microorganisms and were incubated in filter-sterilized human saliva supplemented with 1% sucrose at 37 °C and 5% CO_2 for 67 h (medium changed twice daily). No additional bacterial or fungal cells were inoculated into the saliva-based medium, except those within the initially attached biostructures. The biofilm structural organization on the tooth-enamel surface was assessed via a multilabelling approach. Bacterial EPS glucans were labeled via supplementing the saliva medium with 1 μM Alexa Fluor 647 dextran conjugate. At the end of the experiment, biofilms formed on the tooth-enamel surface were stained using Syto9 (for *S. mutans*) and Concanavalin A-tetramethylrhodamine (for *C. albicans*). The 3D biofilm structure formed on the tooth-enamel surface was imaged using a 20× water-immersion objective (numerical aperture = 1.0) on the Zeiss LSM800 system. Amira software (version 5.4.1) was used to generate 3D renderings of the biofilm architecture.

Enamel surface analyses. To assess the enamel structural damage and mineral loss, we conducted multi-scale surface analyses after removing the biofilms from the tooth enamel (15, 16). In brief, after biofilm imaging, the biomass was removed using enzymatic treatment (dextranase and mutanase) followed by water bath sonication, which was optimized for biofilm removal without causing artificial surface damage (15). Macroscopically, the demineralized areas on tooth-enamel surfaces (similar to those found clinically in severe childhood tooth-decay) were visualized using a stereomicroscope (Zeiss AxioZoom V16). Then, the surface topography and roughness of the tooth-enamel surface were assessed by a non-destructive confocal topography analysis using a 50× (numerical aperture = 0.95) objective on the Zeiss LSM800 microscope following previously reported protocols (15). The 3D microtopography datasets were processed using ConfoMap software (Zeiss) to generate surface properties and 3D surface renderings. Next, the tooth-enamel specimens were mounted on acrylic rods and sectioned (100 ± 20 thickness) with a hard tissue microtome (Silverstone-Taylor Hard Tissue Microtome, Series 1000 Deluxe) for transversal microradiography. The sections were placed in the TMR-D system and x-rayed at 45 kV and 45 mA at a fixed distance, for 12 s. An aluminum step wedge was X-rayed under identical conditions. The digital images were analyzed using the TMR software v.3.0.0.18, with sound enamel defined at 87% mineral volume (17).

References for SI Methods

1. J. Xiao, *et al.*, *Candida albicans* carriage in children with severe early childhood caries (S-ECC) and maternal relatedness. *PloS One* **11**, e0164242 (2016).

2. M. L. Falsetta, *et al.*, Symbiotic relationship between *Streptococcus mutans* and *Candida albicans* synergizes virulence of plaque biofilms *in vivo*. *Infect. Immun.* **82**, 1968–1981 (2014).

3. H. Koo, P. L. Rosalen, J. A. Cury, Y. K. Park, W. H. Bowen, Effects of compounds found in propolis on *Streptococcus mutans* growth and on glucosyltransferase activity. *Antimicrob. Agents Chemother.* **46**, 1302–1309 (2002).

4. D. Kim, *et al.*, Spatial mapping of polymicrobial communities reveals a precise biogeography associated with human dental caries. *Proc. Natl. Acad. Sci.* **117**, 12375–12386 (2020).

5. D. Kim, H. Koo, Spatial design of polymicrobial oral biofilm in its native disease state. *J. Dent. Res.* **99**, 597–603 (2020).

6. A. Simon-Soro and Z. Ren, *et al.* Polymicrobial aggregates in human saliva build the oral biofilm. mBio. **13**, e0013122 (2022).

7. J. Xiao, *et al.*, *Candida albicans* and early childhood caries: a systematic review and meta-analysis. *Caries Res.* **52**, 102–112 (2018).

8. W. H. Bowen, R. A. Burne, H. Wu, H. Koo, Oral biofilms: pathogens, matrix, and polymicrobial interactions in microenvironments. *Trends Microbiol.* **26**, 229–242 (2018).

9. Z. Ren, *et al.*, Dual-targeting approach degrades biofilm matrix and enhances bacterial killing. *J. Dent. Res.* **98**, 322–330 (2019).

10. R. Singh, *et al.*, Affordable oral health care: dental biofilm disruption using chloroplast made enzymes with chewing gum delivery. *Plant Biotechnol. J.* **19**, 2113–2125 (2021).

11. J. Autio-Gold, The role of chlorhexidine in caries prevention. *Oper. Dent.* **33**, 710–716 (2008).

12. M. E. L. Baldino, *et al.*, Nystatin effect on chlorhexidine efficacy against *Streptococcus mutans* as planktonic cells and mixed biofilm with *Candida albicans*. *Clin. Oral Investig.*, 1–10 (2021).

13. A. J. Paula, G. Hwang, H. Koo, Dynamics of bacterial population growth in biofilms resemble spatial and structural aspects of urbanization. *Nat. Commun.* **11**, 1–14 (2020).

14. R. Hartmann, *et al.*, Quantitative image analysis of microbial communities with BiofilmQ. *Nat. Microbiol.* **6**, 151–156 (2021).

15. Y. Liu, *et al.*, Topical ferumoxytol nanoparticles disrupt biofilms and prevent tooth decay *in vivo* via intrinsic catalytic activity. *Nat. Commun.* **9**, 2920 (2018).

16. D. Kim, *et al.*, *Candida albicans* stimulates *Streptococcus mutans* microcolony development via cross-kingdom biofilm-derived metabolites. *Sci. Rep.* **7**, 1–14 (2017).

17. J. Xiao, *et al.*, Biofilm three-dimensional architecture influences *in situ* pH distribution pattern on the human enamel surface. *Int. J. Oral Sci.* **9**, 74–79 (2017).

SI Figures

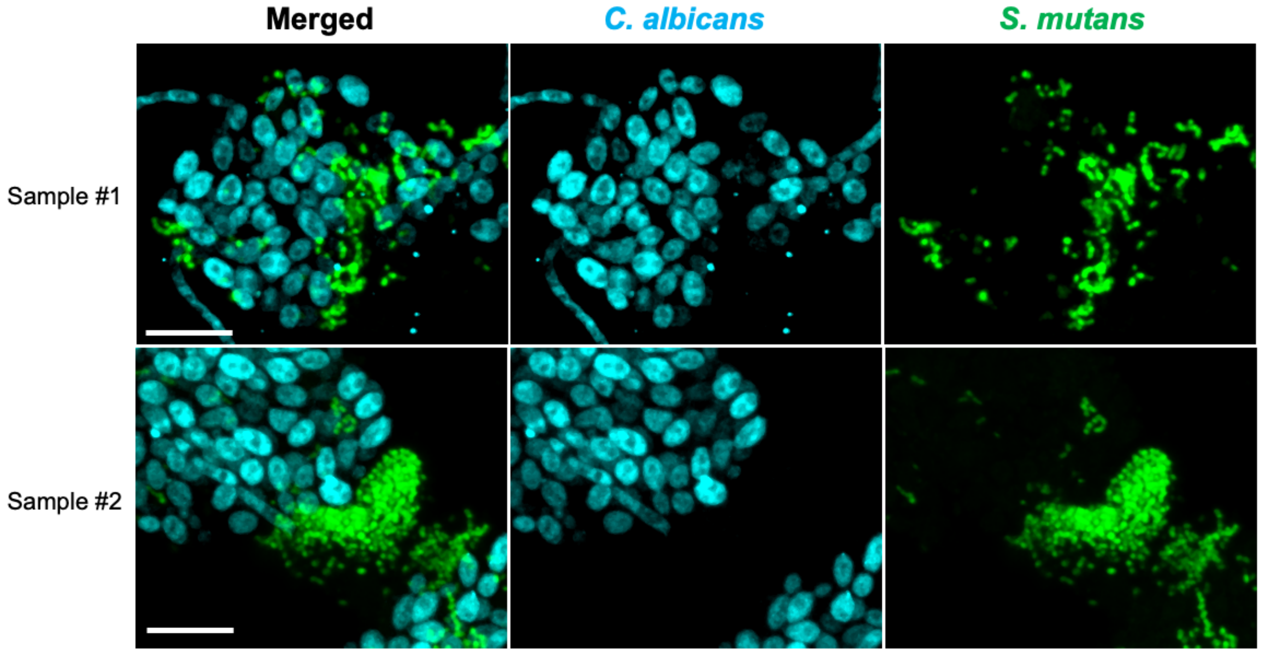

Figure S1. Fluorescence *in situ* hybridization of native-state interkingdom assemblages in saliva from patients with early childhood caries. Saliva from children with severe childhood caries was collected and the naturally-present microbial structure was analyzed by super-resolution confocal imaging and fluorescence *in situ* hybridization (FISH) using species-specific probes (two examples are shown). Saliva from patients was enriched with assemblages comprised primarily of *S. mutans* clusters (green) which were physically associated with *C. albicans* cells (cyan) in yeast and hyphal/pseudo-hyphal forms, building a multicellular structure in the saliva fluid. Scale bar, 10 μm.

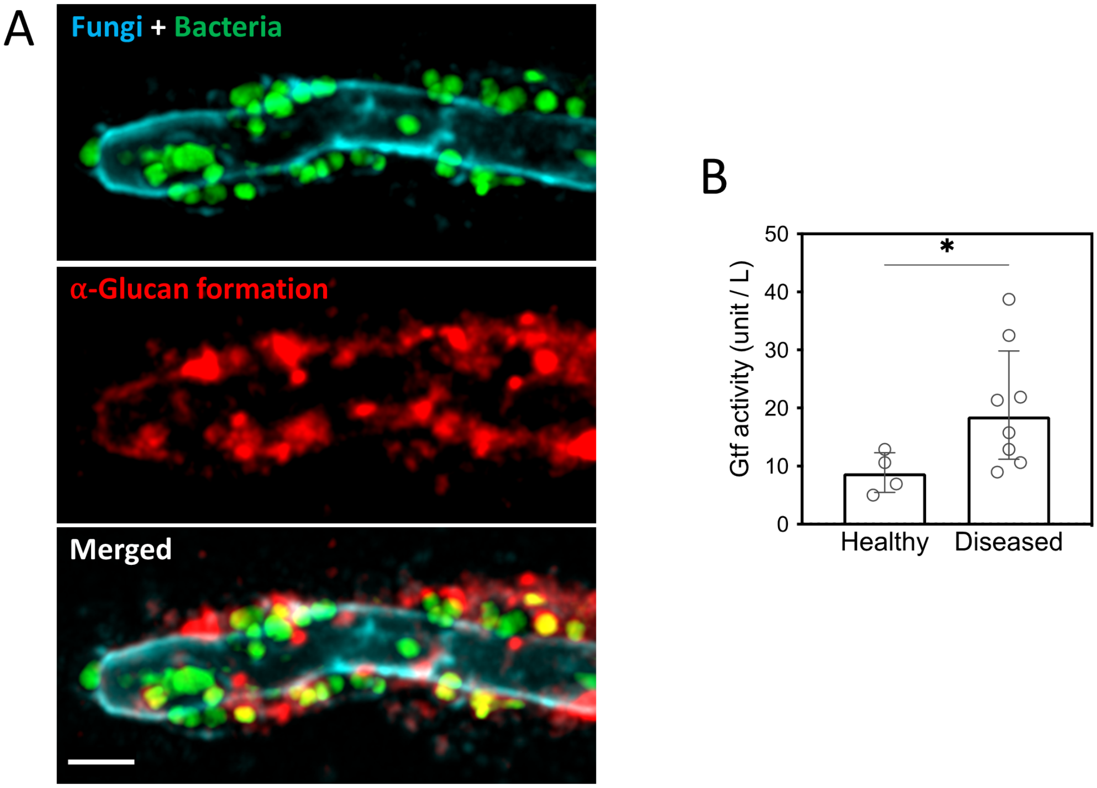

Figure S2. EPS α-glucans formed on the bacterial and fungal cell surfaces of the interkingdom assemblage and glucosyltransferases (Gtf) activity in saliva. (A) α-Glucans were found on the microbial surfaces of the assemblages in the saliva from patients with early childhood caries using an *ex vivo* glucan detection assay. Cyan, fungal hypha; green, bacteria; red, EPS α-Glucans. Scale bar, 2 μm. (B) Total Gtf enzyme activity in the saliva from healthy (caries-free) and diseased children determined by radiolabeling and scintillation counting. The total Gtf activity in saliva (containing *S. mutans*-derived Gtfs and Gtfs from other species) was measured. Data are presented as median with interquartile range ($N \geq 4$). *, $p < 0.05$ by Mann-Whitney test.

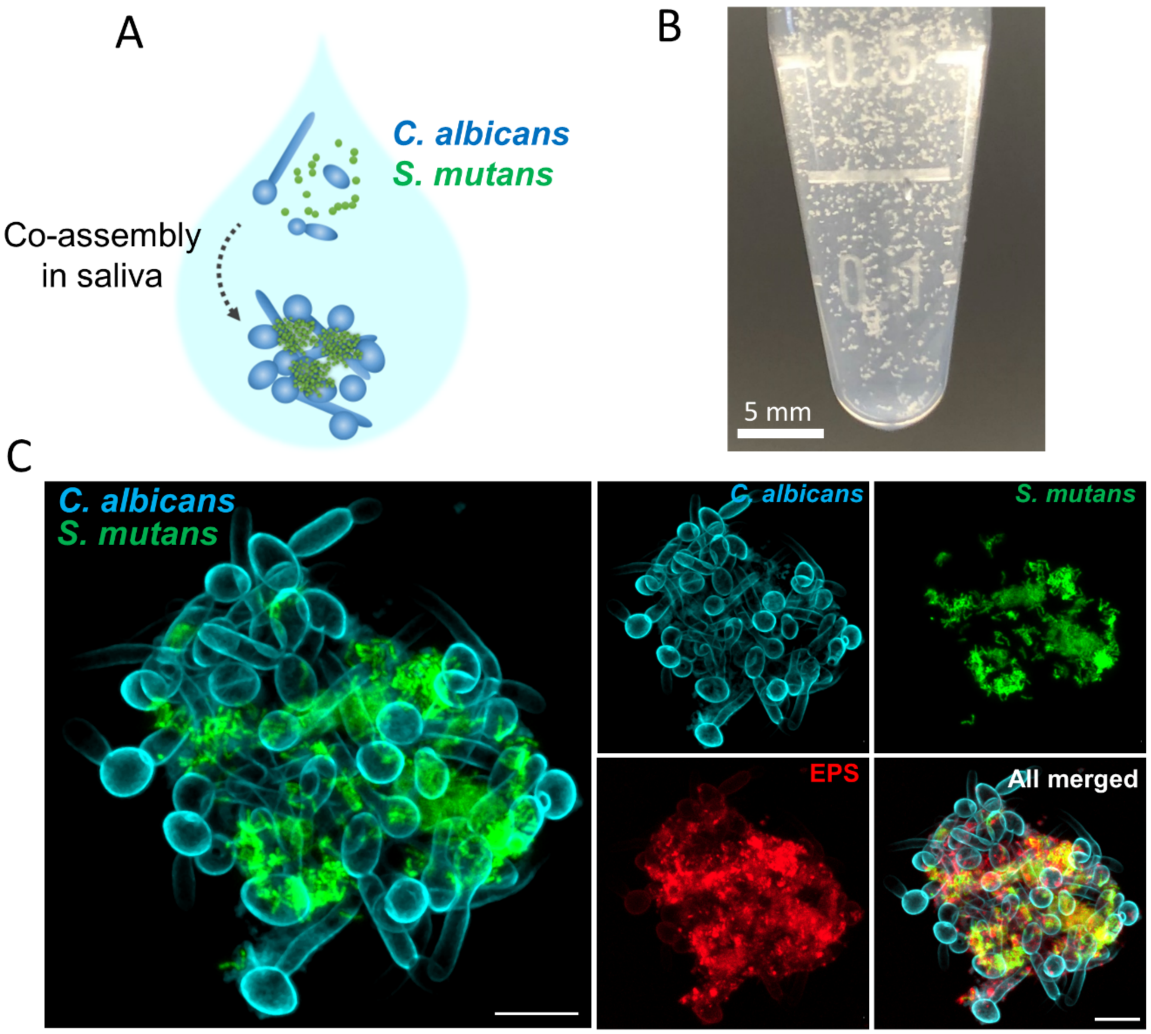

Figure S3. Interkingdom assemblages formed by *C. albicans* and *S. mutans* in human saliva using an experimental model. (A) A schematic diagram illustrating the formation of interkingdom assemblages using pooled human saliva and planktonic *C. albicans* and *S. mutans* cells. (B) Interkingdom assemblages formed by *C. albicans* and *S. mutans* in human saliva are visible as white granules. (C) Super-resolution confocal images of the assemblage formed in the experimental model. *C. albicans* and *S. mutans* can form assemblages with similar structural features to those naturally present in the patients' saliva, which are characterized by *Candida* yeast and hyphal forms (in cyan) intertwined with *S. mutans* clusters (in green) and EPS α-glucans (in red). Scale bar, 10 μm.

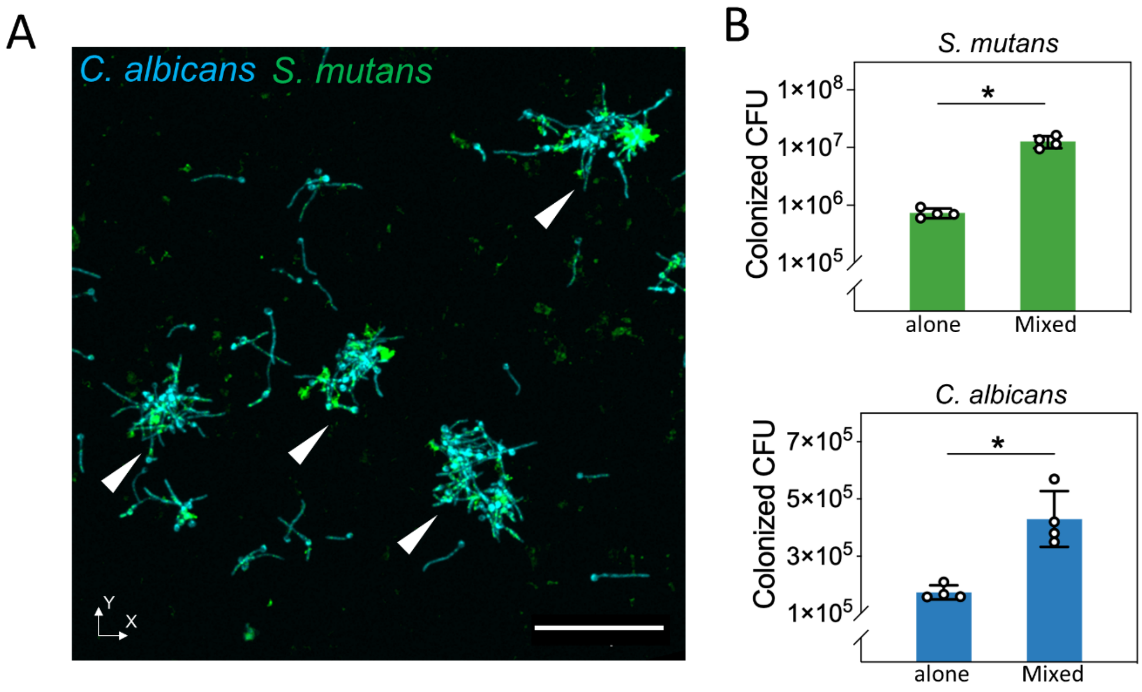

Figure S4. *C. albicans* and *S. mutans* can co-colonize a tooth-mimetic hydroxyapatite surface as a pre-assembled unit. (A) Confocal image (top view) of the saliva-coated hydroxyapatite (sHA) surfaces with colonized fungi and bacteria. The interkingdom assemblages formed in the saliva prior to surface contact can attach to the sHA surface as individual colonizing units (arrowheads). Cyan, *C. albicans*; green, *S. mutans*. Scale bar, 100 μm. (B) CFU measurements of *S. mutans* and *C. albicans* attached on the sHA surface. A defined population of fungi and bacteria (either 10^5 CFU/mL *C. albicans*, or 10^7 CFU/mL *S. mutans*, or a mixture: 10^5 CFU/mL *C. albicans* and 10^7 CFU/mL *S. mutans*) was used as the inoculum. We found higher counts of *S. mutans* (top) and *C. albicans* (bottom) attached on the surface when they colonized as assemblages versus each species alone in saliva. *, $p < 0.05$ by Student's *t*-test.

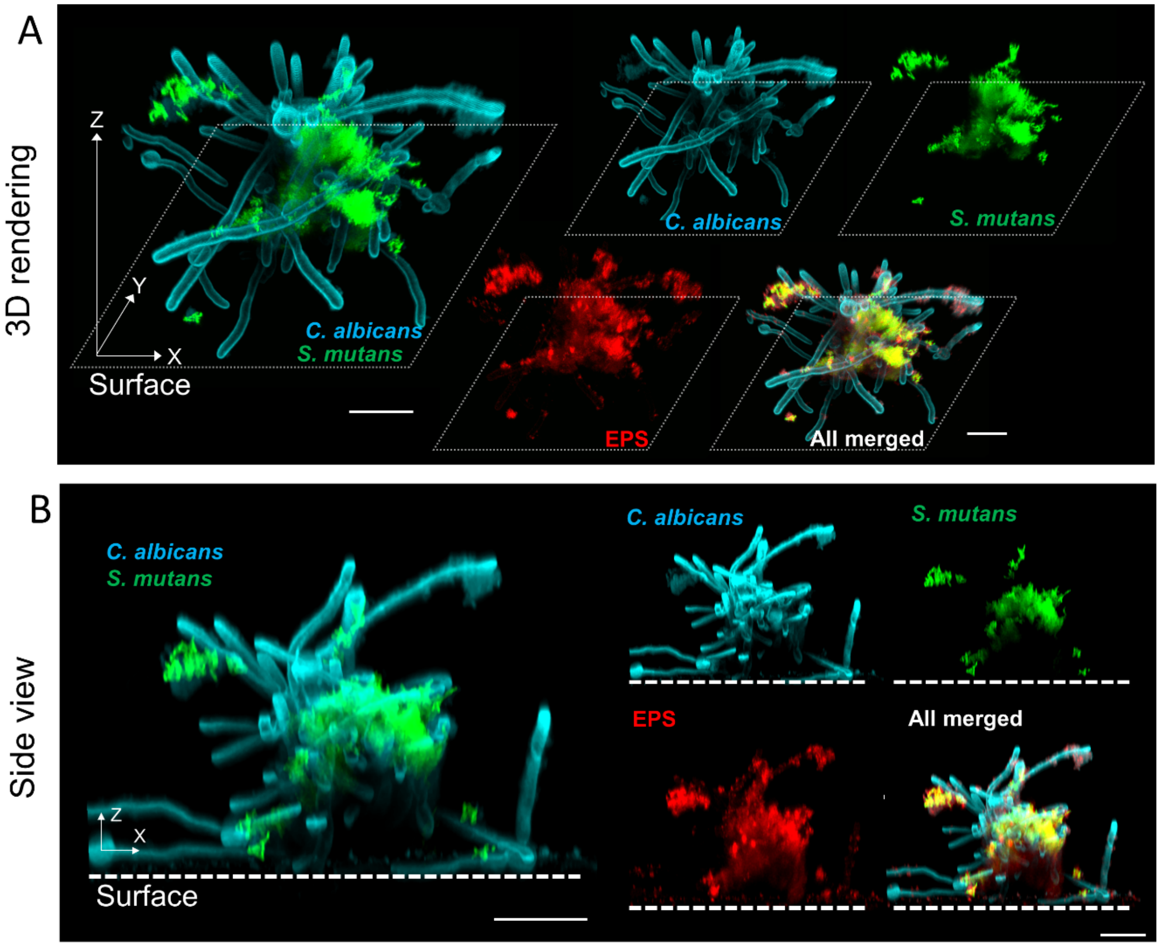

Figure S5. Confocal fluorescence image (3D and orthogonal reconstruction) of interkingdom assemblage attached on the surface. (A) Three-dimensional image reveals a network of *C. albicans* yeasts and hyphae (in cyan) harboring *S. mutans* clusters (in green) in the assemblage structure, which attaches to the sHA surface (dotted line) as a multicellular group. Both the fungi and bacteria are enmeshed by EPS α-glucans (in red). (B) Orthogonal reconstruction shows *C. albicans* hyphae (in cyan) located at the periphery adhering to the surface (dotted line), whereas most of the bacterial clustered (in green) were attached onto the fungal surface like 'cargo'. Scale bars, 20 μm.

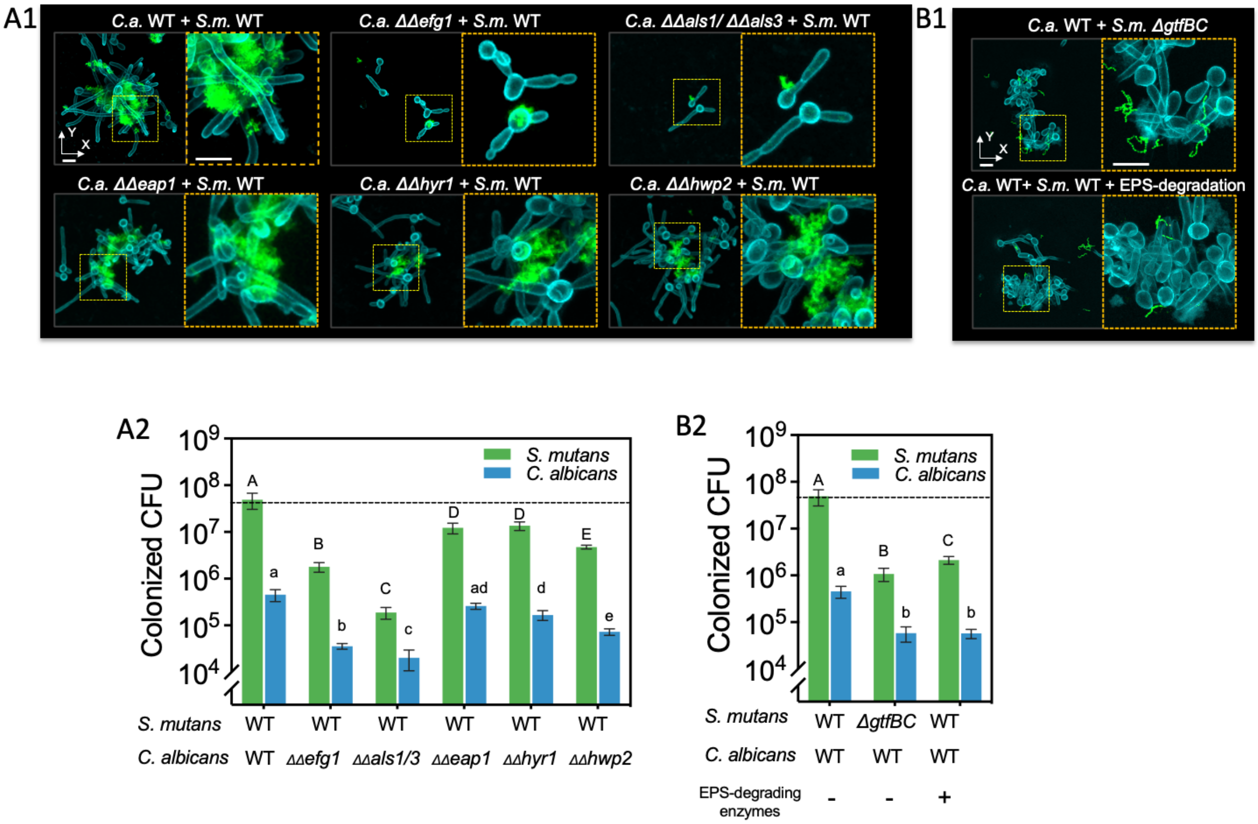

Figure S6. Surface colonization of interkingdom assemblages formed by *C. albicans* or *S. mutans* mutant strains, and with EPS α-glucans degradation. (A1) Confocal image of surface-attached assemblages formed by *C. albicans* mutant strains (in cyan) and *S. mutans* wild type (in green), and (A2) CFU counts of both species recovered from the surface. (B1) Confocal image of surface-attached assemblages formed by *C. albicans* wild type strain with *S. mutans* double-mutant of *gtfB* and *gtfC*, and the assemblage formed by wild type *C. albicans* and *S. mutans* in the presence of EPS-degrading enzymes (dextranase and mutanase), and (B2) CFU counts of both species recovered from the surface. For all the tested combinations, a defined population (10^5 CFU/mL for *C. albicans* and 10^7 CFU/mL for *S. mutans*) was used as the inoculum. Groups that do not share an uppercase letter (for *S. mutans*) or a lowercase letter (for *C. albicans*) are significantly different ($p < 0.05$) by one-way analysis of variance with Tukey's multiple-comparison test). Abbreviation: *C.a.*, *C. albicans*; *S.m.*, *S. mutans.* Scale bars, 10 μm.

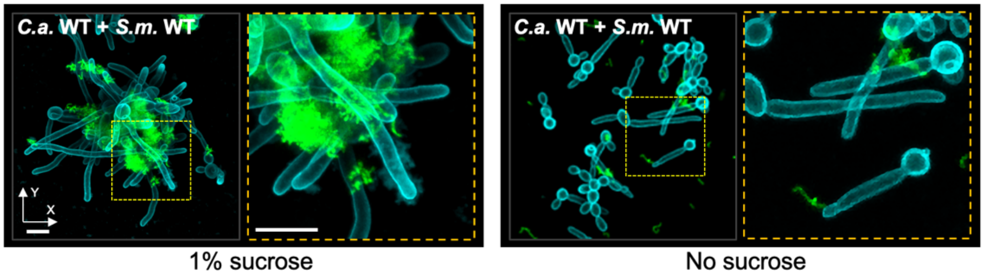

Figure S7. Surface colonization of interkingdom assemblages formed by *C. albicans* and *S. mutans* in the absence of sucrose. Confocal image of surface-attached assemblages formed by wild type *C. albicans* (in cyan) and *S. mutans* (in green) in the presence or absence of 1% sucrose. In the absence of sucrose, the substrate for streptococcal Gtf enzymes to synthesize EPS α-glucans, the ability of *C. albicans* and *S. mutans* to colonize as structured interkingdom assemblages was impaired. Abbreviation: *C.a.*, *C. albicans*; *S.m.*, *S. mutans.* Scale bars, 10 μm.

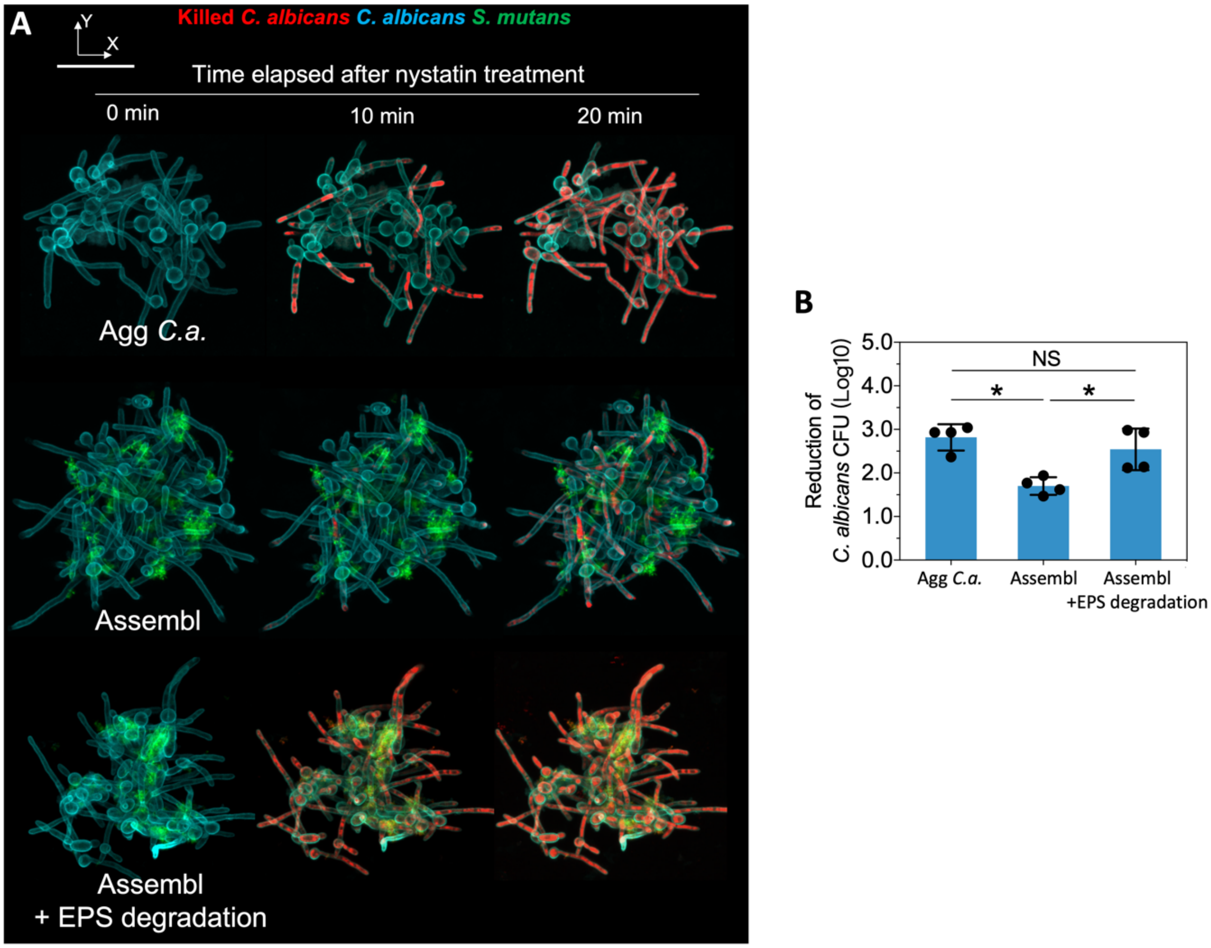

Figure S8. Nystatin treatment of intact interkingdom assemblages. (A) Time-lapse confocal image of antifungal killing. Fungal cells killed by nystatin (250 μg/mL) are visualized using Toto-3 iodide (in red). Left image illustrates biostructures prior to antifungal exposure (0 min; green, *S. mutans*; cyan, *C. albicans*). Images in the middle and on the right show the real-time killing profile (merged channels) within the same surface-attached biostructure after 10 min and 20 min of exposure, respectively. Nystatin was unable to effectively kill fungal cells within the intact assemblages. Abbreviations: Agg *C.a.*, aggregated *C. albicans*; Assembl, interkingdom assemblage. Scale bar, 20 μm. (B) Reduction of *C. albicans* cell viability after 20-min antifungal treatment using nystatin (250 μg/mL). *, $p < 0.05$ by one-way analysis of variance with Tukey's multiple-comparison test.

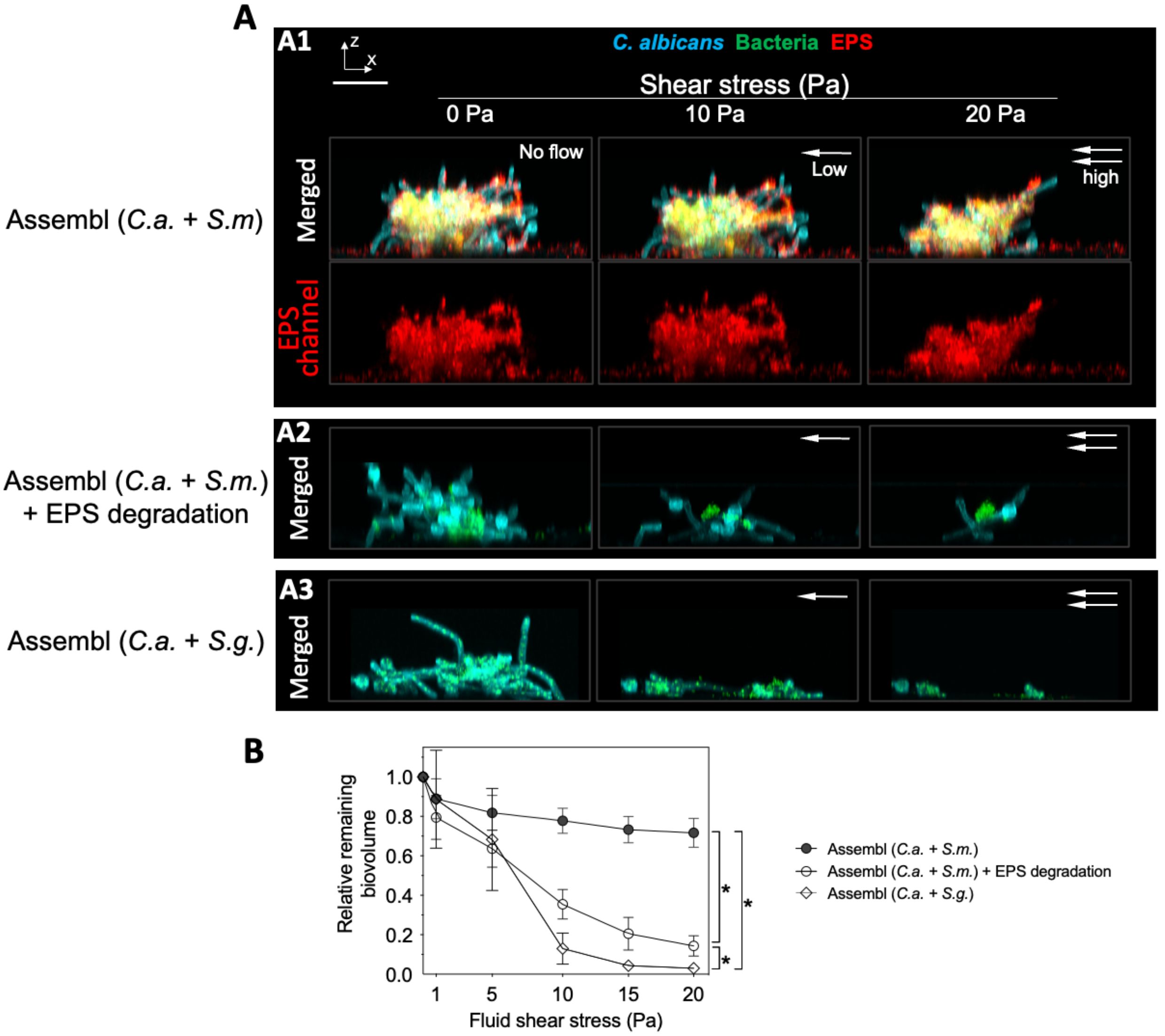

Figure S9. Shear stress resistance of surface-attached interkingdom assemblage. (A1) Time-lapse confocal image of the cellular response of an intact, EPS-enmeshed interkingdom assemblage (formed by *C. albicans* and *S. mutans*) to the surface detachment force. Left images (0 Pa) illustrates a surface-attached assemblage prior to shear stress exposure. Middle and right images show the same assemblage after exposure to intermediate shear stress (10 Pa) or high shear stress (20 Pa), respectively. Top, merged image; bottom, EPS fluorescence channel only. Color scheme: Green, *S. mutans*; cyan, *C. albicans*; red, EPS α-glucans. (A2) Surface-attached assemblage (*C. albicans* and *S. mutans*) was readily removed by increased fluid shear after α-glucan matrix degradation using exogenously supplied glucanohydrolases (dextranase and mutanase). Color scheme: Green, bacteria (*S. mutans* or *S. gordonii*); cyan, *C. albicans*; red, EPS α-glucans. (A3) Surface-attached assemblage (*C. albicans* and *S. gordonii*) was readily removed by increased fluid shear. Abbreviations: Assembl, interkingdom assemblage; *C.a.*, *C. albicans*; *S.m.*, *S. mutans*; *S.g.*, *S. gordonii*. Scale bar, 20 μm. (B) Relative remaining biovolume of the surface-attached assemblages (*C. albicans*-*S. mutans* assemblage with or without EPS degradation and *C. albicans*-*S. gordonii* assemblage) after applying different shear stress. The remaining biovolume was calculated as the total biovolume of bacteria (*S. mutans* or *S. gordonii*) and *C. albicans* on the surface. *, $p < 0.05$ by one-way analysis of variance with Tukey's multiple-comparison test.

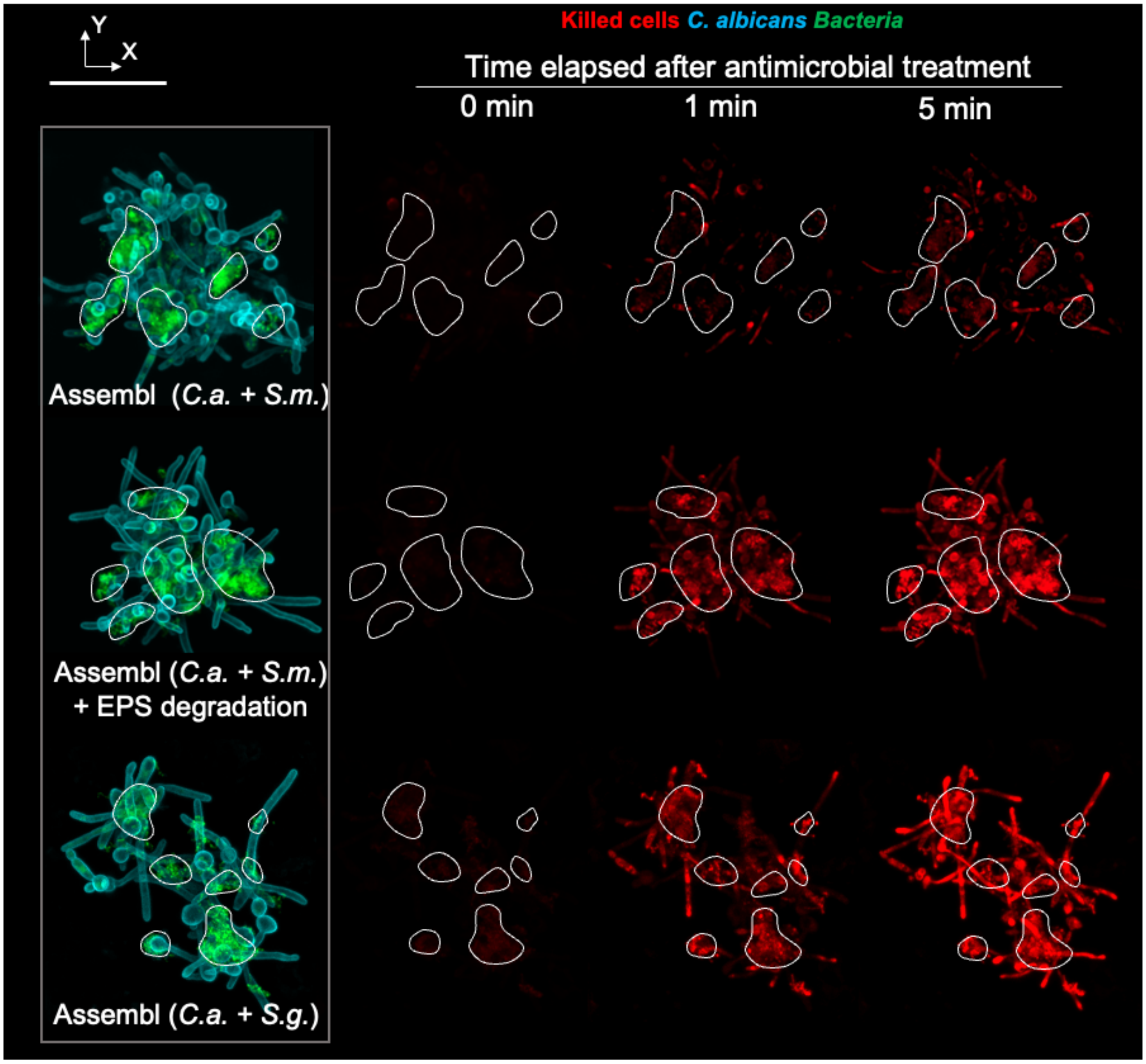

Figure S10. Antimicrobial tolerance of surface-attached interkingdom assemblage. Time-lapse confocal image showing antimicrobial killing by chlorhexidine (100 μg/mL); dead cells (bacteria and fungi) are visualized using TOTO-3 iodide (in red). Left image in the box illustrates *C. albicans*-*S. mutans* assemblage (with and without EPS degradation by dextranase and mutanase) or *C. albicans*-*S. gordonii* assemblage prior to antimicrobial exposure. Images on the right show the real-time killing profile (red channel only) within the same surface-attached assemblage. White solid lines indicate the bacterial clusters within interkingdom assemblages. The data show that the microbes in the *C. albicans*-*S. mutans* assemblage (after EPS α-glucan degradation) and in the *C. albicans*-*S. gordonii* assemblage were effectively and homogeneously killed by chlorhexidine (100 μg/mL). Color scheme: Green, bacteria (*S. mutans* or *S. gordonii*); cyan, *C. albicans;* red, dead cells (bacteria and *C. albicans*). Abbreviation: Assembl, interkingdom assemblage; *C.a.*, *C. albicans*; *S.m.*, *S. mutans*; *S.g.*, *S. gordonii*. Scale bar, 20 μm.

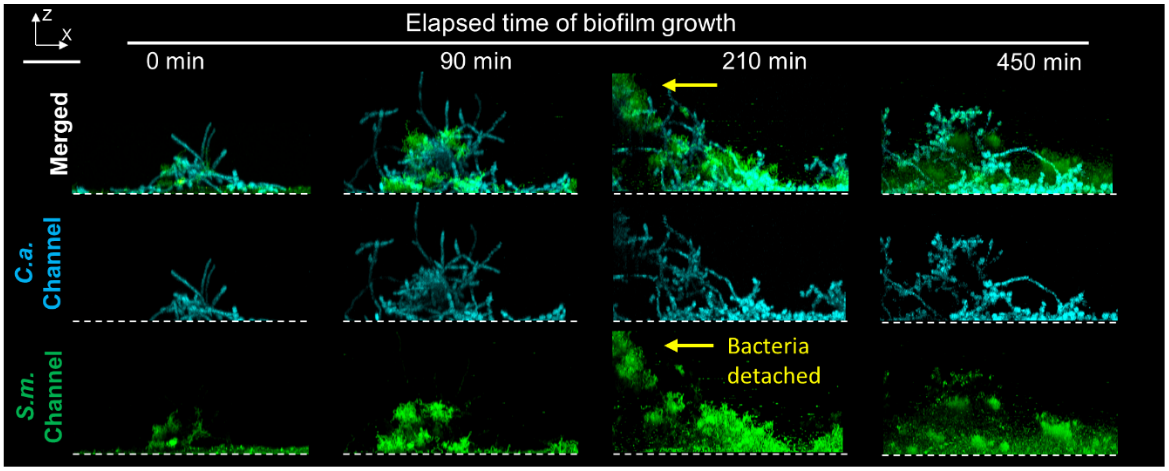

Figure S11. Disruption of bacterial attachment to the *Candida* hyphae and the "hitch-hiking" growth behavior. Orthogonal projections of time-lapse confocal images of growing interkingdom assemblages with continuous EPS α-glucan degradation. Glucanohydrolases (dextranase and mutanase) were continuously provided in the culture medium flow which causes degradation of EPS α-glucan within the surface-attached assemblage, preventing new glucan synthesis and accumulation. In these conditions, bacterial attachment to the *Candida* hyphae was impaired despite growth of both species, and therefore the bacterial cells could not "hitch-hike" on the developing fungi, suggesting that α-glucan degradation could disrupt this mode of mobility. Color scheme: Green, *S. mutans*; cyan, *C. albicans*. Yellow arrow, bacteria clusters detached from the fungi during the growth. Abbreviation: *C.a.*, *C. albicans*; *S.m.*, *S. mutans*. Scale bar, 50 μm.

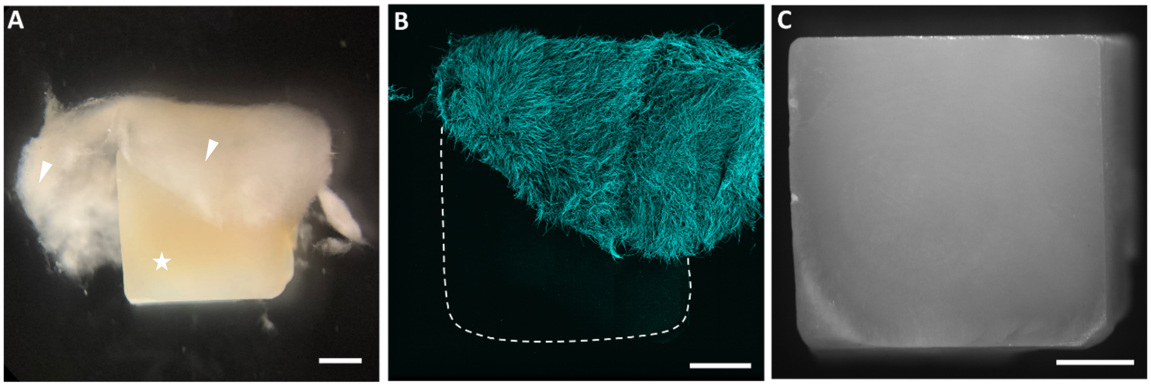

Figure S12. Fungal biofilm formed on tooth enamel surface by *C. albicans* without co-assembly with bacteria. (A) Aggregated *C. albicans* alone as inoculum was able to attach and develop into a biofilm (arrowheads) on the human enamel surface (asterisk) but detached after gentle dip-washing. (B) Confocal image of the biofilm formed by aggregated *C. albicans* on the same human enamel surface. Cyan, *C. albicans*. Dotted line, outline of the human tooth enamel specimen. (C) Enamel surface underneath the biofilm, imaged using brightfield stereomicroscopy. Macroscopic inspection could not detect caries lesions or demineralization on the tooth surface, indicating no significant enamel damage; Scale bar, 1 mm.

Legends for Movies

Movie S1 (separate file). Aggregated *S. mutans* alone remained in their initial position on the surface during the growth. Scale bar, 50 μm.

Movie S2 (separate file). In the interkingdom assemblage, bacterial clusters were lifted away from the surface and transported laterally while continuously growing along with fungi, thus “hitch-hiking” on the elongating hyphae. A forward-leaping motion was observed during the growth of the interkingdom assemblage. The fungal network rapidly protruded forward to move the biostructure laterally. Green, *S. mutans*; cyan, *C. albicans.* Scale bar, 50 μm.

Movie S3 (separate file). Specific deactivation of fungal growth in the interkingdom assemblage using nystatin impaired fungal filamentation and resulted in complete loss of “hitch-hiking” mobility. Green, *S. mutans*; cyan, *C. albicans.* Scale bar, 50 μm.
